# Regulation of cerebral cortical neurogenesis by the Pax6 transcription factor

**DOI:** 10.3389/fncel.2015.00070

**Published:** 2015-03-10

**Authors:** Martine N. Manuel, Da Mi, John O. Mason, David J. Price

**Affiliations:** Centre for Integrative Physiology, The University of Edinburgh, EdinburghUK

**Keywords:** proliferation, cell cycle, differentiation, neuronal fate, neurotransmitter fate, cortical lamination, BAF complex, Meis2

## Abstract

Understanding brain development remains a major challenge at the heart of understanding what makes us human. The neocortex, in evolutionary terms the newest part of the cerebral cortex, is the seat of higher cognitive functions. Its normal development requires the production, positioning, and appropriate interconnection of very large numbers of both excitatory and inhibitory neurons. Pax6 is one of a relatively small group of transcription factors that exert high-level control of cortical development, and whose mutation or deletion from developing embryos causes major brain defects and a wide range of neurodevelopmental disorders. Pax6 is very highly conserved between primate and non-primate species, is expressed in a gradient throughout the developing cortex and is essential for normal corticogenesis. Our understanding of Pax6’s functions and the cellular processes that it regulates during mammalian cortical development has significantly advanced in the last decade, owing to the combined application of genetic and biochemical analyses. Here, we review the functional importance of Pax6 in regulating cortical progenitor proliferation, neurogenesis, and formation of cortical layers and highlight important differences between rodents and primates. We also review the pathological effects of *PAX6* mutations in human neurodevelopmental disorders. We discuss some aspects of Pax6’s molecular actions including its own complex transcriptional regulation, the distinct molecular functions of its splice variants and some of Pax6’s known direct targets which mediate its actions during cortical development.

## Introduction

The expansion of the cerebral cortex is a major hallmark of mammalian evolution, particularly in the primate lineages where it achieves its greatest complexity in humans (Martin, 1990). Despite great variation in size, there are many similarities in the structure and function of the cerebral cortex across all mammalian species. These similarities have encouraged neuroscientists to use relatively simple cortices, such as those of rodents, as models in which to investigate biological processes and mechanisms with likely relevance to humans. The strength of rodent models, in particular mice, derives in large part from the genetic and transgenic approaches that can be used to study molecular mechanisms. Much of the research described in this review comes from studies of mice and the view of cortical development and Pax6’s role in that process presented here is strongly biased toward mouse corticogenesis. It is important to recognize, however, that significant differences exist between the developing and mature cortices of primate and non-primate species that are more than just differences of scale, and we shall highlight some of these. It is remarkable that many high-level regulatory genes such as *Pax6* are themselves extremely highly conserved between primates and non-primates although they influence and control aspects of cortical development that differ: for example, the amino acid sequences of human PAX6 and mouse Pax6 are identical (Ton et al., 1992) although Pax6’s expression in primate embryos extends to cortical structures that do not exist in mice. This implies that evolutionary changes in the functions of these regulators have been achieved by changes in the mechanisms regulating their expression and in the ways in which the downstream molecular networks respond to them, but we know almost nothing about these evolutionary changes at a molecular level.

The cerebral cortex is derived from the dorsal component (or pallium) of the embryonic telencephalon, which is itself the anterior-most subdivision of the forebrain (Kiecker and Lumsden, 2005). The cerebral cortex can be further sub-divided into distinct regions, including the neocortex, a novel acquisition of mammals that has evolved between the phylogenetically older archicortex (comprising entorhinal cortex, retrosplenial cortex, subiculum, and hippocampus) and paleocortex (olfactory piriform cortex). The evolutionary expansion of the neocortex is thought to account for much of the increase in overall brain size and complexity in more advanced species (Krubitzer and Kaas, 2005; O’Leary et al., 2007). The neocortex (hereafter referred to simply as cortex) contains an extraordinarily large number of neurons arrayed in a six-layered sheet, with neurons in each layer organized into a complex network of local circuits and subcortical connections. In primates, some of these layers are subdivided in some cortical areas: for example, in primary visual cortex, layer 4 is subdivided into layers 4a, b, and c. The primate cortex is also characterized by an expansion of the superficial layers of the cortex, layers 2 and 3 (also known as the supragranular layers), which have an important function in the transfer of information between cortical areas. Increased intracortical information processing is likely to have contributed to heightened cognitive ability. In all mammals, the cortex comprises two major groups of neurons: the majority are excitatory glutamatergic projection neurons (70–80%), which exhibit a characteristic pyramidal morphology and extend axons to distant intracortical, subcortical, and subcerebral targets; a minority are inhibitory GABAergic non-pyramidal interneurons (25–30% in primates, 15–20% in rodents), which have short axons and project locally (Hendry et al., 1987; Beaulieu, 1993). An appropriate balance between the excitatory and inhibitory circuitry of the cortex is critical for its normal function.

*Pax6* is a pivotal gene in CNS development. It is expressed when the major components of the developing CNS first emerge after neural tube closure and its expression patterns change considerably as major structures such as the cerebral cortex specialize and expand. Its expression plays a major role in the subsequent development of the regions that continue to express it. In this review we shall discuss how Pax6 plays critical roles in aspects of corticogenesis that include the early patterning of telencephalic subdivisions, control of cortical cell number, normal cortical layer formation and the development of the correct balance of excitatory and inhibitory neurons. We shall review what is known about the upstream control of *Pax6*’s transcription, the molecular basis of its functions and its actions on genes downstream of it and the cellular processes they regulate.

## Corticogenesis in Rodents and Primates and the Cortical Expression of Pax6

The closure of the neural tube is accompanied by its disproportionate expansion anteriorly to generate the early forebrain. The earliest cortical progenitors undergo symmetric proliferative divisions at the ventricular surface to amplify a pool of progenitors, an increasing proportion of which then divide asymmetrically to regenerate progenitors and to produce other cell types including neurons.

**FIGURE 1:**
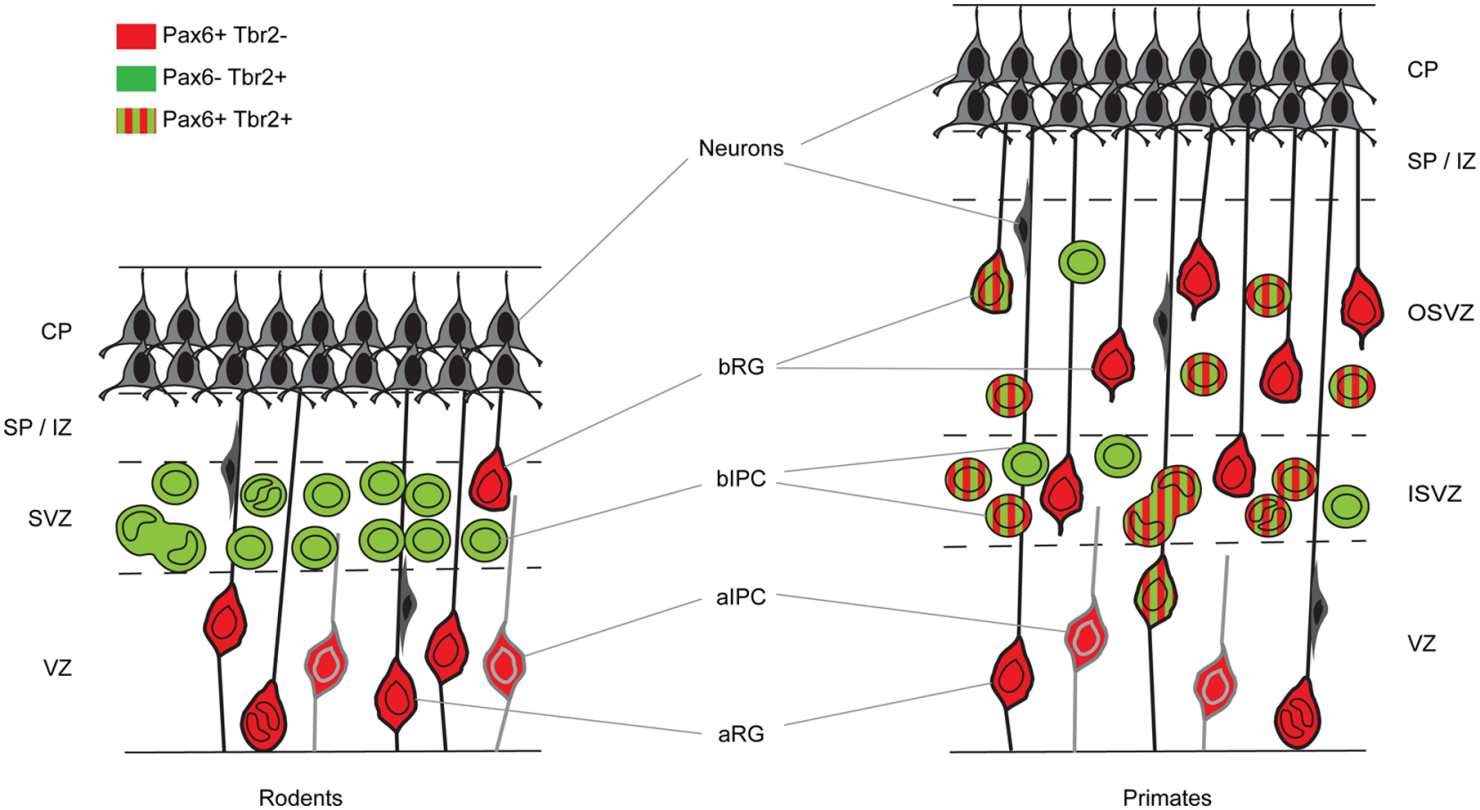
Cortical germinal areas of rodents and primates. Coronal section through the developing cortex of a rodent and a primate showing progenitor types and whether they express Pax6 and/or Tbr2. CP, cortical plate; SP, subplate; IZ, intermediate zone; SVZ, subventricular zone; ISVZ, inner subventricular zone; OSVZ, outer subventricular zone; VZ, ventricular zone; aRG, apical radial glia; bRG, basal radial glia; aIPC, apical intermediate progenitor cell; bIPC, basal intermediate progenitor cell.

In mouse, the generation of excitatory cortical neurons occurs between embryonic day 11 (E11) and E18 (Gillies and Price, 1993; Price et al., 1997; Levers et al., 2001). Their progenitors are located in one of two pallial germinal epithelia, namely the ventricular zone (VZ), which lies adjacent to the ventricles, and the subventricular zone (SVZ), which lies just above the VZ (**Figure 1**). The early symmetrically dividing cortical neuroepithelial cells (NECs) that each produce two daughter NECs per division and whose population rapidly expands laterally and radially, transform into another type of progenitor called, for historical reasons, radial glial cells (RGCs; **Figure 1**). RGCs, whose long processes span the neuroepithelium, have been known for a long time to provide guidance for migrating neurons (Levitt and Rakic, 1980; Rakic, 1988; Hatten, 2002; Tan and Shi, 2013). Despite having morphological and molecular features associated with glial cells, they are progenitors capable of generating other types of progenitor, neurons and glial cells (Malatesta et al., 2000, 2003; Miyata et al., 2001; Noctor et al., 2001; Skogh et al., 2001; Heins et al., 2002; Tan and Shi, 2013). RGCs constitute the majority of the VZ progenitor population. They are often referred to as apical progenitors (APs) due to the location of their cell body close to the ventricular or apical surface of the VZ, to which they extend a short process while sending a longer basal process radially to the pial surface of the cortex (Cameron and Rakic, 1991; Bentivoglio and Mazzarello, 1999; Tan and Shi, 2013). As they progress through the cell cycle, RGCs undergo interkinetic nuclear migration. Their nucleus migrates radially through the cytoplasm such that mitosis occurs at the apical ventricular surface and S-phase at a basal position in the VZ. While at early stages of corticogenesis RGCs predominantly self-renew via symmetric divisions, they progressively switch to asymmetric divisions, which produce one daughter RGC and one daughter cell with a heightened level of commitment (Noctor et al., 2001, 2004; Haydar et al., 2003; Huttner and Kosodo, 2005; Tan and Shi, 2013). The differentiating daughter cell either migrates radially to the pial surface and differentiates into a neuron or migrates to the SVZ to become an intermediate progenitor cell (IPC), also called a basal progenitor (BP) or basal IPC (bIPC; **Figure 1**; Haubensak et al., 2004; Miyata et al., 2004; Noctor et al., 2004). BPs accumulate in the SVZ and divide mainly symmetrically to generate two neurons. It is thought that most cortical projection neurons are generated by BPs (Farkas and Huttner, 2008). In addition to RGCs, the VZ contains another smaller subpopulation of APs called short neural precursors (SNPs), also known as apical IPCs (aIPC; **Figure 1**). Unlike RGCs, aIPCs do not self-renew and only generate pairs of neurons via symmetric divisions (Gal et al., 2006; Tan and Shi, 2013).

Significant progress in understanding the mechanisms underlying corticogenesis has been made through analysis of gene expression in both progenitor cells and post-mitotic neurons. Such studies have revealed a crucial role for transcription factors (TFs) as molecular markers of distinct progenitor and neuron types and as key regulators of progenitor cell proliferation and cell fate decisions. At the onset of neurogenesis, TFs including Pax6, Emx2, and Tlx expressed in the cortical neuroepithelium function mainly to influence areal patterning and regulate progenitor cell proliferation (Bishop et al., 2000, 2002; Muzio et al., 2002; Muzio and Mallamaci, 2003; Stenman et al., 2003a,b; Hevner, 2006). As neurogenesis proceeds, a large number of TFs are involved in regulating the balance between progenitor cell proliferation and neuronal differentiation. Regulating this balance is essential for the generation of the correct proportions of different classes of neurons and subsequent circuit assembly.

**FIGURE 2:**
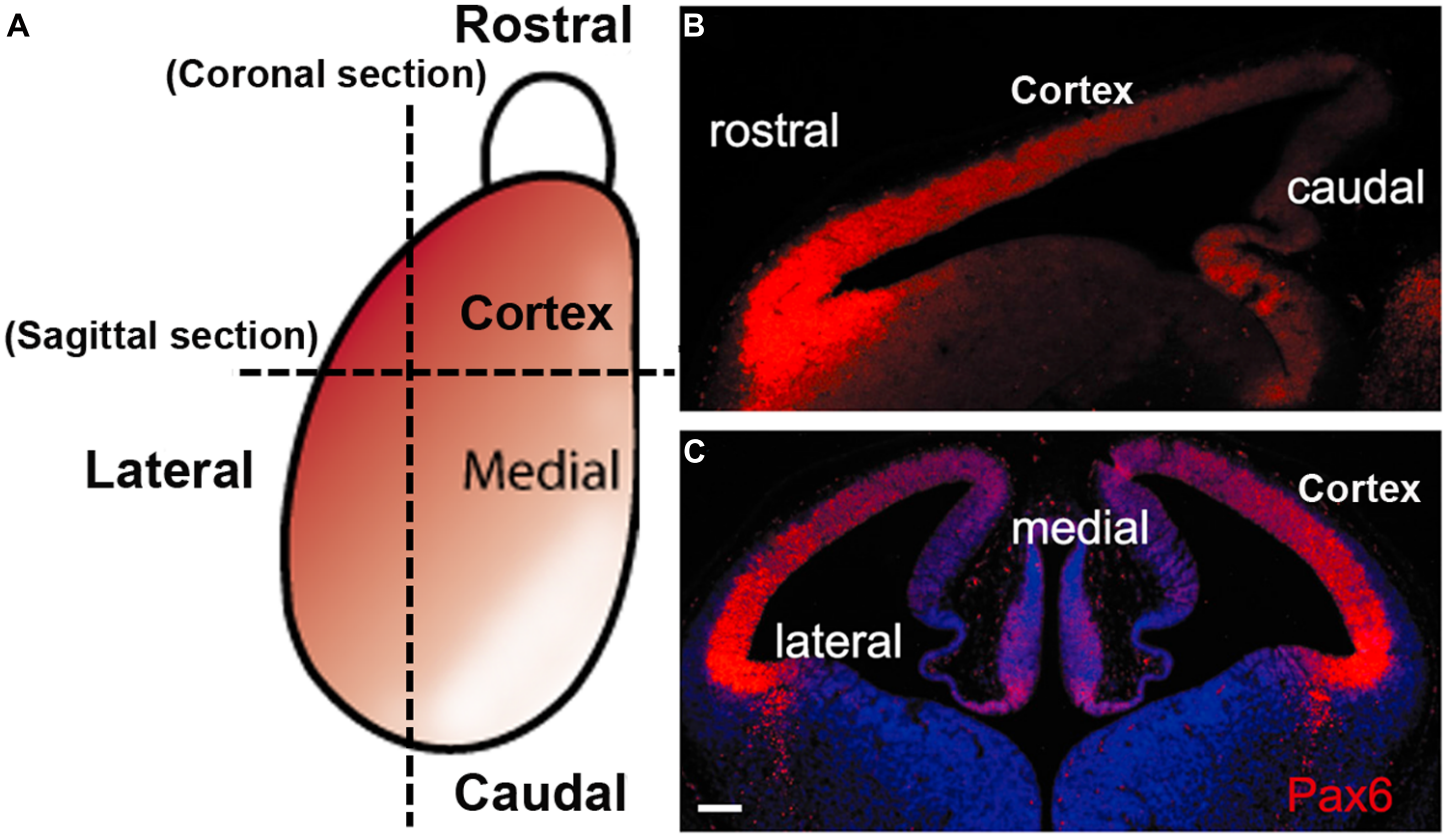
The gradient expression pattern of Pax6 protein in the mouse developing cortex. **(A)** A schematic diagram shows the Pax6 protein (red) is normally expressed in a ^high^rostrolateral to ^low^caudomedial gradient in the developing cortex during early cortical development. **(B,C)** immunofluorescence staining for Pax6 protein in sagittal section **(B)** and coronal section **(C)** from E12.5 mouse WT embryos (scale bars, 100 μm).

**FIGURE 3:**
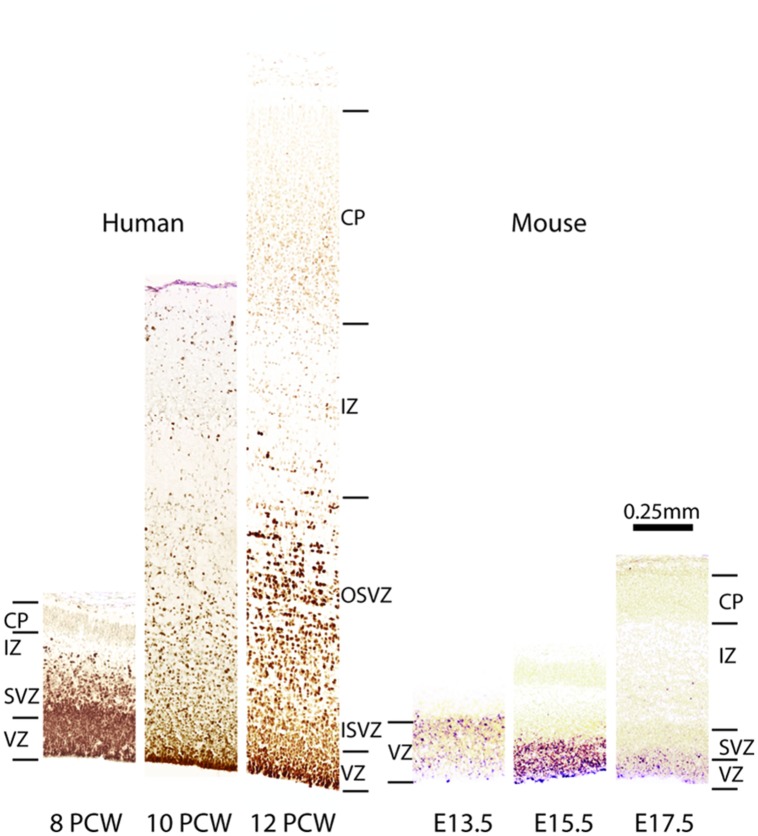
Comparison of Pax6 expression in embryonic human and mouse cortex. Images from human cortices at 8, 10, and 12 post-coital weeks (PCWs) were generated using material from the Human Developmental Biology Resource (www.hdbr.org) as part of the HuDSeN (Kerwin et al., 2010) human gene expression spatial database (http://www.hudsen.org) based at Newcastle University. PAX6 is expressed in the ventricular zone (VZ) and subventricular zone (SVZ) at 8 PCW. The SVZ divides into an outer and an inner subventricular zone (OSVZ and ISVZ), both of which continue to express PAX6. In mice, Pax6 expression is confined almost exclusively to the VZ during corticogenesis, as shown here at embryonic days (E) 13.5, 15.5, and 17.5. Additional abbreviations: CP, cortical plate; IZ, intermediate zone.

During the neurogenic period in the mouse cortex, Pax6 is expressed by VZ APs in a high rostro-lateral to low caudo-medial gradient (**Figures 2** and **3**). As well as being graded spatially, the expression of Pax6 is also graded temporally, with highest levels present at the onset of corticogenesis. APs that give rise to BPs transiently express the proneural TF Neurogenin 2 (Ngn2; Britz et al., 2006). Pax6 is not expressed in BPs which are characterized by their expression of another TF, Tbr2, or in post-mitotic neurons which express Tbr1 (**Figure 1**; Englund et al., 2005). Thus a sequential Pax6 → Ngn2 → Tbr2 → Tbr1 expression correlates with the transition of APs to BPs to post-mitotic neurons.

In the macaque cortex, Rakic (1974) showed that cortical neurogenesis occurs between E45 and E100. Gestation in this species lasts 165 days and so cortical neurons are generated relatively earlier in gestation in primates than in rodents. The primate SVZ forms earlier and shows a much greater expansion than in rodents, becoming the predominant progenitor zone by mid-corticogenesis (Smart et al., 2002). It is split into the inner and outer SVZs separated by an inner fiber layer (Smart et al., 2002; Lukaszewicz et al., 2005; Zecevic et al., 2005; Fietz et al., 2010; Hansen et al., 2010; Florio and Huttner, 2014). The inner subventricular zone (ISVZ) contains mainly IPCs similar to those in the rodent SVZ whereas the outer subventricular zone (OSVZ) contains mainly basal RGCs (bRGCs) similar to the APs present in the rodent VZ (**Figure 1**). bRGCs undergo proliferative divisions and self renewing asymmetric divisions to generate one bRGC daughter cell and one IPC that can proliferate further (Fietz et al., 2010; Hansen et al., 2010; Florio and Huttner, 2014). bRGCs have also been observed in the rodent SVZ but, while they constitute about half of all progenitors present in the primate SVZ, they account for only a minute fraction of the SVZ progenitors in rodents (**Figure 1**). The OSVZ is the major source of supragranular layer neurons (Letinic et al., 2002; Lukaszewicz et al., 2005).

The sequential Pax6 → Ngn2 → Tbr2 → Tbr1 expression that correlates with the AP → BP → post-mitotic neuron transition in mice is not found during primate corticogenesis. In contrast to rodents, Pax6 is expressed by progenitors in the VZ, ISVZ, and OSVZ in primates (**Figure 3**; Fietz et al., 2010; Betizeau et al., 2013; Florio and Huttner, 2014) and many progenitors co-express Pax6 and Tbr2 (**Figure 1**). In the macaque, the majority of VZ progenitors (60–80%) express only Pax6 during early and mid-stages of corticogenesis (up to E79), but at later stages (after E79) 40% of them co-express Tbr2 (Betizeau et al., 2013; Florio and Huttner, 2014; **Figure 1**). In the ISVZ, 60–80% of progenitors co-express Pax6 and Tbr2, 5–30% express only Tbr2 and less than 15% express only Pax6. In the OSVZ, Pax6, and Tbr2 are co-expressed by 25–50% of progenitors while 20–35% express only Pax6 and 10–20% only Tbr2 (**Figure 1**; Betizeau et al., 2013; Florio and Huttner, 2014).

In mice, cortical GABAergic interneurons originate from distant germinal domains in the ganglionic eminences and follow tangential migratory routes to reach the developing cortex (Gelman and Marin, 2010). In primates, the origin of cortical interneurons is controversial. It has been proposed that while many interneurons have a ventral telencephalic origin, a significant fraction are produced in the progenitor layers of the cortex itself during the second half of corticogenesis (Letinic et al., 2002; Zecevic et al., 2005; Petanjek et al., 2008; Jakovcevski et al., 2011). However, a recent study by Hansen et al. (2013) found no evidence of interneuron production in the cortical wall. Instead, their analysis suggests that, as in rodents, the vast majority of human cortical interneurons are produced in the ganglionic eminences (Hansen et al., 2013).

In all mammalian species, the positions adopted by neurons migrating from the cortical progenitor zones to the overlying developing cortical plate (CP) are related to their birthdate. Each successive generation of newly born projection neurons bypasses earlier-born neurons and settles close to the pial edge of the CP. Thus, cortical layers (with the exception of layer 1) are formed in a deep-first superficial-last sequence (Angevine and Sidman, 1961; Berry and Rogers, 1965; Rakic, 1974; McConnell, 1995; Tan and Shi, 2013). When projection neurons arrive in their final laminar positions, they undergo terminal differentiation including elaboration of their dendrites and axons to establish connections and eventually form the cortical circuitry. Projection neurons in each layer tend to exhibit similar gene expression patterns, morphologies and organization of afferent and efferent connections (Stiles and Jernigan, 2010).

## Human Brain Disorders Associated with *PAX6* Mutations

In humans, heterozygous loss-of-function mutations of *PAX6* cause sight-threatening congenital eye defects, typically including severe hypoplasia of the iris (aniridia) and retina. These mutations are also associated with a range of neurological and psychiatric conditions including nystagmus, impaired auditory processing and verbal working memory, autism, and mental retardation (Malandrini et al., 2001; Bamiou et al., 2004, 2007a,b; Davis et al., 2008; Hingorani et al., 2009; Maekawa et al., 2009). These conditions are linked to structural brain defects including reduced size of the corpus callosum and anterior commissure, abnormalities of the cerebral cortex and cerebellum and absence of the pineal gland (Sisodiya et al., 2001; Free et al., 2003; Mitchell et al., 2003; Bamiou et al., 2004, 2007b; Ellison-Wright et al., 2004).

**FIGURE 4:**
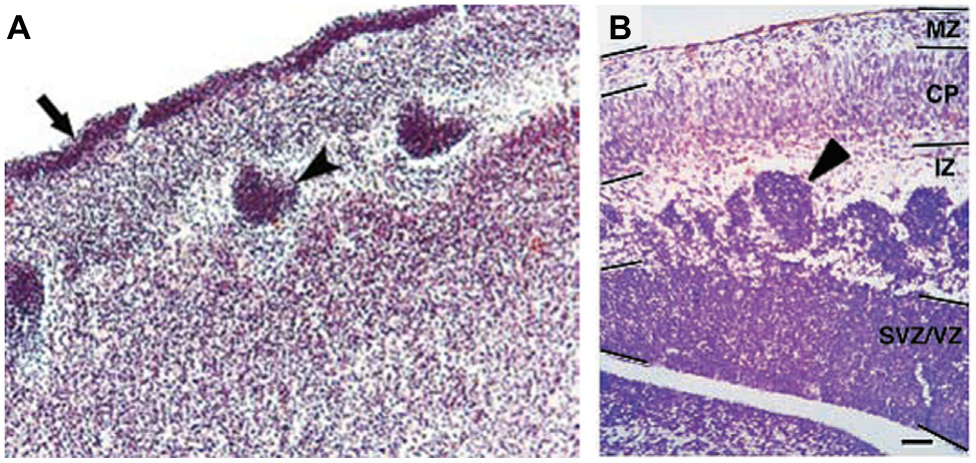
Histology of *Pax6^-/-^* developing cortex in human and mouse. **(A)** Coronal section through the cortex of a fetus with a compound heterozygosity for two *PAX6* mutations showing a layer of germinal cells on the surface of the cortex (arrow) and heterotopia of germinal cells within the cortex (arrowhead). Photograph taken from Schmidt-Sidor et al. (2009). **(B)** Coronal section through the cortex of a *Pax6^-/-^* mutant mouse embryo showing clusters of germinal cells in the intermediate zone (arrowhead). Photograph from Caric et al. (1997).

Only four cases of children with mutations in both *PAX6* alleles (compound heterozygotes) have been reported (Glaser et al., 1994; Schmidt-Sidor et al., 2009; Solomon et al., 2009). Two of them survived past birth, one only for about 1 week (Glaser et al., 1994), the other until at least 4 years (Solomon et al., 2009). The former had anophthalmia, the latter microphthalmia, and both had numerous defects in the CNS including agenesis of the corpus callosum and microcephaly. Glaser et al. (1994) described a disturbed stratification of the cerebral cortex with heterotopic islands of germinal and ependymal cells, as well as focal polymicrogyria of the cerebral cortex. Two other cases were sibling fetuses with the pregnancies terminated at 21 and 23 weeks (Schmidt-Sidor et al., 2009). In both cases the brain was very small, filling only 1/3 of the cranial cavity, and displayed completely disorganized structures of the brain hemispheres and cerebellum. Microscopic analysis showed that the cerebral hemispheres contained an enormous amount of germinal matrix both in the inner parts and at the surface of the hemispheres. The cortex was very narrow with a paucity of cells with an irregular distribution of large neurons. The structure of the cortex was disturbed with a thick layer of germinal cells on its surface, a poorly defined marginal zone and no normal stratification. In the entire cortex the cells were mainly in clusters. The development of the white matter was also severely disturbed. The intermediate zone was absent. In several places of the cortex and also between clusters of neuroblasts inside the brain hemispheres there were fascicles of axons which did not form normal tracts. The heterotopia of germinal cells observed in the human compound heterozygotes (**Figure 4A**) are reminiscent of the clusters of germinal cells found in the intermediate zone of the cortex of *Pax6^-/-^* mouse embryos (**Figure 4B**; Caric et al., 1997). In these Pax6 mutant embryos it is thought that the clusters form as a consequence of a cell non-autonomous migration defect (Caric et al., 1997).

## Structure and Transcriptional Regulation of the *Pax6* Locus

**FIGURE 5:**
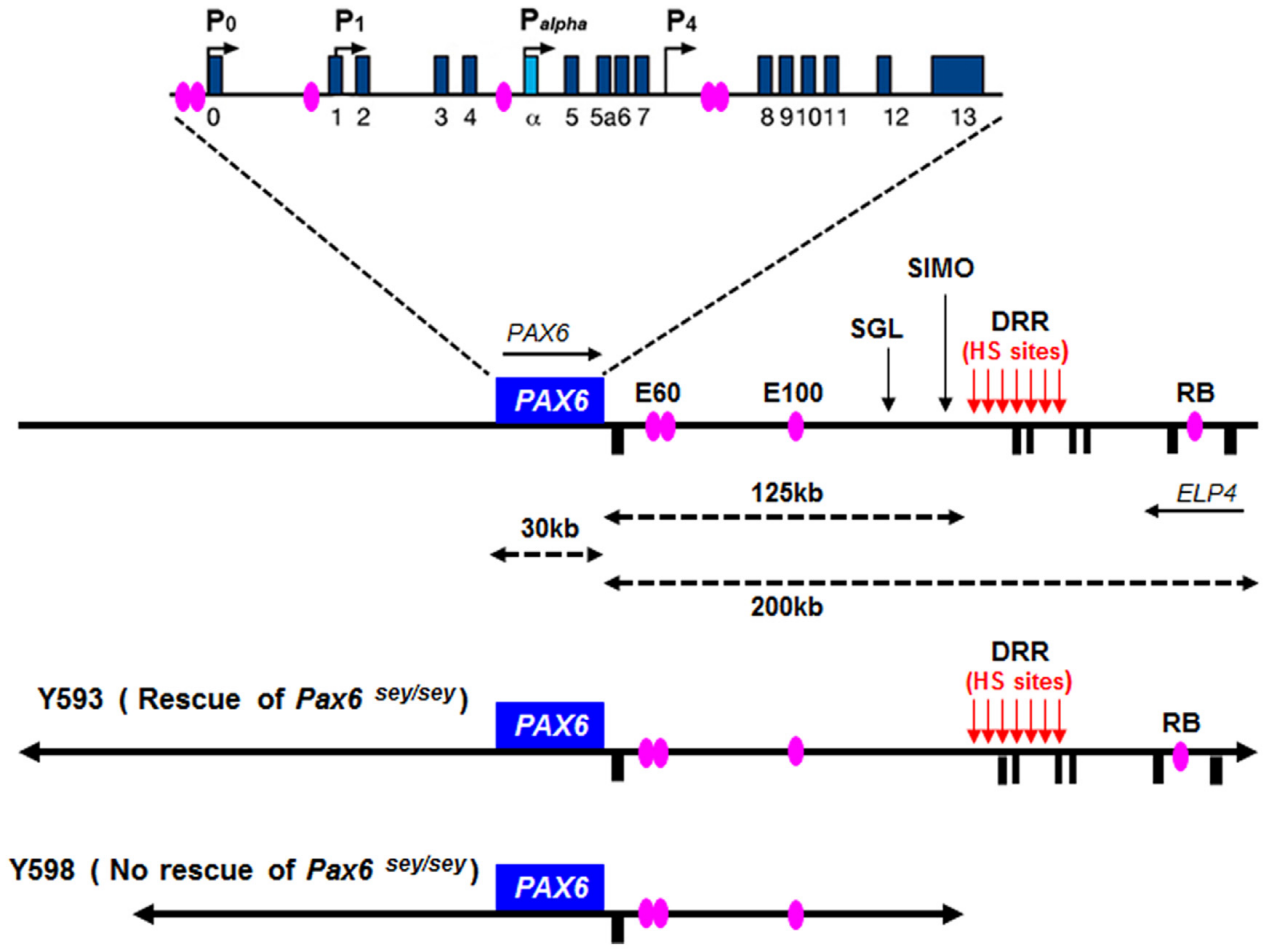
Structure and transcriptional regulation of the *PAX6* locus. Schematic map of human chromosome 11p13 shows the locations of the PAX6 and ELP4 loci, which are in an antisense orientation relative to each other. The locations of PAX6 promoters (P0, P1, Pa, and P4) are indicated by black arrows. Blue rectangles indicate *PAX6* exons, while black rectangles below the line indicate *ELP4* exons. The known highly conserved regulatory elements, such as E60, E100 and RB, are indicated by purple ellipses. The breakpoints of the two distal-most aniridia-associated rearrangements are indicated by “SGL” and “SIMO.” The location of the downstream regulatory region (DRR) is shown by red arrows. The known DNaseI hypersensitive sites (HS sites) within the DRR region are shown by red arrows. The schematic maps of YACs Y593 (420 kb) and Y589 (310 kb) are indicated. In transgenic mice, Y593, but not Y589, rescues the mouse *Sey* phenotype and homozygous *Sey* lethality.

In both human and mouse, the *Pax6* gene has 16 exons distributed over a 30 kb genomic region including alternatively spliced exons *alpha* and 5a (Glaser et al., 1992; Williams et al., 1998; Kammandel et al., 1999; Plaza et al., 1999a; Xu et al., 1999b). Four transcription start sites have been identified in mouse *Pax6*, associated with the P_0_, P_1_, P*_alpha_*, and P_4_ promoters respectively (**Figure 5**; Kammandel et al., 1999; Xu et al., 1999b; Kleinjan et al., 2004; Morgan, 2004). Transcriptional regulation of the *Pax6* locus is particularly complex. A number of short-range regulatory elements have been identified in the vicinity of the *Pax6* coding region which control tissue-specific *Pax6* expression (**Figure 5**; Kammandel et al., 1999; Plaza et al., 1999b; Kleinjan et al., 2001, 2004; Griffin et al., 2002). Some of these elements exhibit overlapping tissue specificity, particularly in the eye, telencephalon and diencephalon, suggesting that they exert functions through cooperative interactions.

Although these short-range regulatory elements account for much of *Pax6*’s normal expression pattern in mice, genetic analyses in both humans and mice revealed that they are insufficient to drive the full pattern of *Pax6* expression, particularly in the eye and forebrain (Kim and Lauderdale, 2006; Kleinjan et al., 2006; McBride et al., 2011). The first evidence that PAX6 expression is influenced by distant regulatory elements located far downstream of *PAX6*’s coding exons came from studies of aniridia patients, a subset of which harbor chromosomal rearrangements whose breakpoints are located far downstream of the *PAX6* transcriptional unit (Fukushima et al., 1993; Fantes et al., 1995; Lauderdale et al., 2000; Kleinjan et al., 2001; Crolla and Van Heyningen, 2002). The most distant of these breakpoints, designated “SIMO," is located 124 kb downstream of the *PAX6* polyadenylation site (Fantes et al., 1995; Kleinjan et al., 2001; **Figure 5**). The functional importance of regulatory regions around distant breakpoints was confirmed by the finding that a yeast artificial chromosome (YAC) comprising 420 kb of the human *PAX6* coding sequence and flanking regions stretching beyond the SIMO breakpoint rescues the mouse *small eye* (*Sey*) phenotype, whereas a YAC transgene with 110 kb less DNA sequence at the 3^′^ end fails to rescue (Kleinjan et al., 2001). Subsequent analyses revealed the presence of essential 3^′^ distant regulatory elements within a 75 kb region termed the *PAX6* downstream regulatory region (DRR) located 3^′^ of the SIMO breakpoint (**Figure 5**; Schedl et al., 1996; Kleinjan et al., 2001; McBride et al., 2011). A number of other conserved regulatory elements, including E60, E100, and RB (**Figure 5**), located either upstream or downstream of the DRR region are thought to be crucial for the induction of *PAX6* expression in the eye and forebrain (Griffin et al., 2002; Kleinjan et al., 2002, 2006; Kim and Lauderdale, 2006; McBride et al., 2011).

We recently reported a detailed examination of a series of novel transgenic reporter mice lines, carrying versions of either the full length 420 kb YAC described above or truncated versions that lacked putative regulatory elements. The YAC transgenes were modified by the introduction of a tau-GFP reporter cassette into the first coding exon of *PAX6*, such that GFP expression marks cells in which the transgenes are expressed. We found that regulatory elements that lie outside the 420 kb YAC transgene are required for correct *PAX6* expression in sub-regions of the telencephalon and diencephalon, indicating that recapitulation of the full *PAX6* expression pattern requires even more elements than had previously been thought (Mi et al., 2013b).

The *Pax6* locus is subject to autoregulation – *Pax6* has been shown to regulate its own expression. Putative Pax6 binding sites have been identified in *Pax6* regulatory elements in several species. For example, in *Drosophila*, two intronic enhancer elements containing putative Pax6 binding sites mediate auto-activation of *eyeless* (a *Drosophila Pax6* homolog) in the nervous system and eye (Hauck et al., 1999). These elements may serve the same function in vertebrates as they exhibit significant sequence identity between *Drosophila* and vertebrate species (Chow et al., 1999). In addition, Pax6 directly interacts with putative Pax6-responsive elements within the head surface ectoderm-specific enhancer of mouse *Pax6*, positively regulating its own transcription (Aota et al., 2003). Similarly, Pax6 autoregulation in the forebrain was revealed through the identification of a highly conserved regulatory element (CE2) in intron 7 of mouse *Pax6* (**Figure 5**). Overexpressing either Pax6 or its alternative spliced isoform Pax6(5a) positively autoregulated expression of the endogenous *Pax6* locus in Neuro2D and NIH3T3 cell lines (Pinson et al., 2006). The *Pax6* locus is also subject to negative autoregulation, whereby Pax6 represses transcription of the *Pax6* locus, as shown in the developing telencephalon (Manuel et al., 2007). In summary, these studies highlight the fact that appropriate levels of Pax6 expression are regulated by both positive and negative autoregulation during development.

## Pax6 Regulates Cell Cycle Length and Cell Cycle Exit

Generating the correct number of cortical neurons of the correct types involves changes in both the mode of division of cortical progenitors and length of their cell cycle. As discussed above, cortical progenitors switch progressively from proliferative symmetrical divisions to differentiative asymmetrical divisions during corticogenesis. At the same time, the length of the cell cycle in AP cells in mouse cortex increases from 8 h at the onset of neurogenesis (E10) to 18 h at the end (E18), largely due to lengthening of the G1 phase (Takahashi et al., 1993, 1995; Estivill-Torrus et al., 2002). Subsequent studies showed that G1 lengthening acts to promote the genesis of BP cells which have much longer cell cycle length (around 26 h in E14.5 mouse cortex) due to increase in the G1 length (Arai et al., 2011). In the macaque, AP cell cycle length is much longer than in rodents and it follows a different developmental course (Kornack and Rakic, 1998). It lengthens linearly from 23 h at E40 to 54 h at E60, then shortens linearly to 27 h by E80 (Kornack and Rakic, 1998). As the neurogenic period is 10 times longer than in rodents, macaque cortical progenitors undergo many more rounds of division even though the duration of the cell cycle is longer (Kornack and Rakic, 1998), generating a greater number of cortical cells and a larger cortex.

There is mounting evidence that TFs exert region-specific control of cortical progenitor cell cycle progression. Pax6 is one of a number of TFs that are expressed in distinct gradients across cortical areas (Zaki et al., 2003; Sansom and Livesey, 2009; Salomoni and Calegari, 2010; Georgala et al., 2011a) and several studies have implicated Pax6 in the temporal and spatial control of cell cycle duration in cortical progenitors. Loss of Pax6 during early cortical development (E12.5) in mice *in vivo* led to a shortening of the cell cycle of progenitors coupled with higher proportions of asymmetrical divisions, resulting in a temporary increase in the production of post-mitotic neurons (Warren et al., 1999; Estivill-Torrus et al., 2002; Walcher et al., 2013). Experiments with cultured *Pax6^-/-^* mutant cortical cells showed that they too exhibit accelerated proliferation, indicating that Pax6’s effects on the cell cycle are cell autonomous (Estivill-Torrus et al., 2002). In contrast, at the mid-corticogenesis stage (E15.5) *Pax6^-/-^* progenitor cells proliferated more slowly than wild type controls, showing that the effects of Pax6 on the cell cycle of cortical progenitors are context-dependent.

Gain of function studies also support the idea that Pax6 controls cell cycle progression. For example, forced expression of Pax6 impairs progenitor cell proliferation *in vitro* (Heins et al., 2002; Hack et al., 2004; Cartier et al., 2006). In *PAX77* mice, which express PAX6 protein in its normal pattern but at approximately double the wild type level, there is a reduction in the number of proliferating progenitors in the rostral and central cortex, the areas where levels of Pax6 expression are normally highest (Manuel et al., 2007). Further, APs located in rostral cortical areas proliferate more slowly in these mice (Georgala et al., 2011a,b). Thus, both gain and loss of Pax6 function studies have revealed powerful context-specific effects of Pax6 on the cell cycle of progenitor cells in a variety of cortical areas and stages.

**FIGURE 6:**
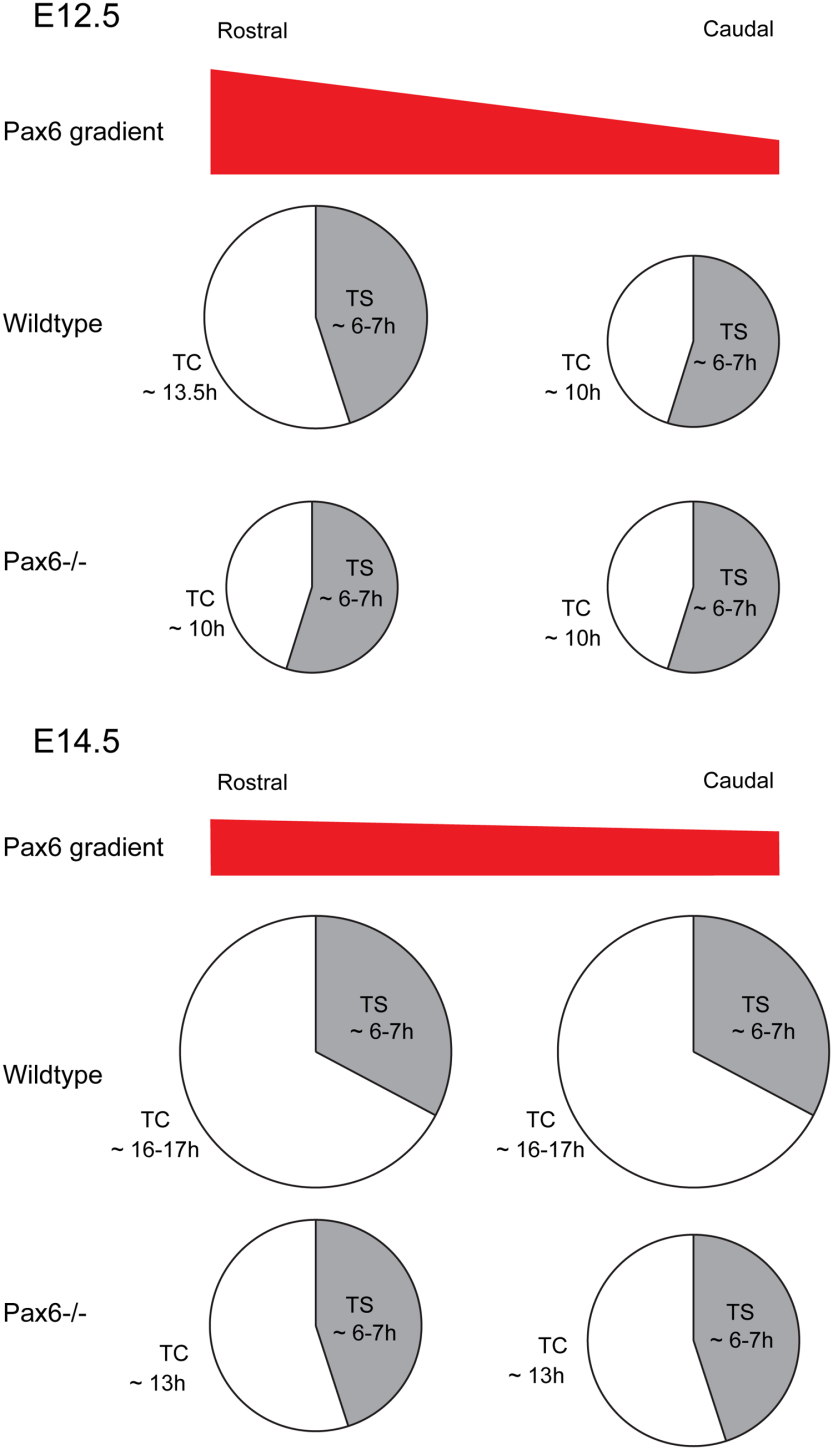
Effect of loss of Pax6 on cortical progenitor cell cycle parameters. Diagram showing the cell cycle length and S-phase length of cortical progenitors at a rostral or caudal position in Wild type or *Pax6^-/-^* embryos. At E12.5 Pax6 is normally expressed in a high rostral to low caudal gradient and loss of Pax6 results in a shortening of the cell cycle rostrally with no effect on S-phase length. At E14.5 Pax6 is expressed in a flatter rostro-caudal gradient and loss of Pax6 causes a shortening of the cell cycle both rostrally and caudally while the S-phase length remains unaffected.

Early in corticogenesis, expression levels of Pax6 vary between different cortical regions but its expression levels become increasingly uniform with embryonic age (Mansouri et al., 1994; Stoykova and Gruss, 1994; Manuel et al., 2007). Some of the evidence cited above indicates that the actions of Pax6 on the cell cycle of cortical progenitors are associated with its expression levels. This assumption is strongly supported by our recent study in which cell cycle parameters were systematically examined in different cortical regions at different developmental stages using mouse models with either constitutive or conditional loss of Pax6 function (Mi et al., 2013a). During early corticogenesis (E12.5), when the Pax6 gradient is steepest, areas of highest expression correlate with regions where the cell cycle duration is longest (**Figure 6**). Loss of Pax6 causes shortening of the cell cycle only in these areas (**Figure 6**). In normal embryos at older ages, the Pax6 gradient becomes progressively more uniform across the cortex, as does cell cycle length, and loss of Pax6 causes shortening of the cell cycle in all areas (**Figure 6**; Mi et al., 2013a). Taken together, these studies indicate that Pax6 primarily exerts a repressive action on the cell cycle progression of cortical progenitors, and distinct expression levels of Pax6 confer its region and age -specific role in regulating progenitor cell proliferation.

As well as regulating the length of the cell cycle in cortical progenitors, Pax6 is involved in the control of cell cycle exit. In the *Pax6^sey/sey^* mutant cortex, the number of progenitor cells that exit the cell cycle during early corticogenesis (E12.5) is increased, leading to a smaller progenitor pool and an increased proportion of newborn neurons (Estivill-Torrus et al., 2002; Quinn et al., 2007). A similar effect has been seen in the eye and the spinal cord, where loss of Pax6 caused precocious neuronal differentiation due to premature cell cycle exit (Philips et al., 2005; Bel-Vialar et al., 2007). Interestingly, a study using *PAX77* mice revealed that increasing Pax6 levels also causes an increase in the proportion of progenitor cells exiting the cell cycle during late corticogenesis (E15.5; Georgala et al., 2011b), indicating that there is no simple dosage-dependent relationship between Pax6 level and progenitor cell cycle exit rate. Recently, we found that loss of Pax6 led to fewer progenitors exiting the cell cycle at the PSPB (pallial–subpallial boundary) at E12.5, the area of the telencephalon where Pax6 expression is highest, contrasting with the previous finding that *Pax6^-/-^* cortical progenitors show increased cell cycle exit at E12.5 (Estivill-Torrus et al., 2002). It appears that, although a general function of Pax6 is to influence cell cycle exit, the exact nature of its effect depends on the molecular and cellular context within which it acts. This context may change corresponding to diverse Pax6 levels in different regions at different developmental stages. In support of this assumption, a number of independent analyses demonstrated that Pax6 exerts different control functions on progenitor proliferation in different cellular contexts: Pax6 enhances proliferation in the neural retina of vertebrates (Marquardt et al., 2001) and *Drosophila* (Dominguez et al., 2004), whereas it inhibits proliferation of human glioblastoma cells (Zhou et al., 2005), cultured corneal epithelial cells (Ouyang et al., 2006) and cortical progenitors in primary cell cultures (Heins et al., 2002; Haubst et al., 2004) as well as *in vivo* (Estivill-Torrus et al., 2002; Berger et al., 2007). Therefore, it is difficult to conclude a simple universally consistent role of Pax6 in the regulation of cell cycle exit.

## Pax6 Direct Targets Regulating Progenitor Cell Proliferation

As described above, Pax6 is important for the regulation of cortical progenitor proliferation (Estivill-Torrus et al., 2002; Manuel et al., 2007; Quinn et al., 2007; Georgala et al., 2011b; Mi et al., 2013a,b). Cell cycle progression is driven by the concerted action of cyclin-dependent kinases (Cdks) and their activating partners, cyclins (Malumbres and Barbacid, 2005). Low levels of Cdk activity are sufficient for cells to transit from G1 into S phase, whereas high-levels of Cdk activity are required for the cell to undergo mitosis (Dehay and Kennedy, 2007; Kaldis and Richardson, 2012). During G1, cells integrate and respond to extracellular cues that either allow the cell to continue cycling or promote its withdrawal from the cell cycle and begin differentiation (Zetterberg et al., 1995). Progression through G1 is mainly driven by the activation of Cdk4/6 and their activating partners, D-type cyclins (Cyclin D1 and D2) as well as Cdk2 and its partner Cyclin E (Malumbres and Barbacid, 2001, 2005). Cdk activity is negatively regulated by members of the cyclin-dependent kinase inhibitors family (CKIs; Polyak et al., 1994; Sherr and Roberts, 1995; Vidal and Koff, 2000). The primary substrate of Cdks during G1 phase is the retinoblastoma protein (pRb). pRb acts as a repressor of E2F TFs through direct interaction. In quiescent cells, the activities of E2Fs are repressed through direct interaction with hypophosphorylated pRb. In cycling cells, mitogenic stimuli that elevate Cyclin levels promote pRb phosphorylation by Cyclin/Cdk complexes, which in turn prevents pRb from binding to E2Fs. Consequently, pRb/E2F complexes are dissociated and free E2Fs are now active and ready to drive the transcription of genes encoding proteins that are involved in G1 phase progression and DNA replication. Gene expression profiling studies showed that Pax6 is capable of regulating the expression of a number of cell cycle genes involved in G1 to S phase transition, such as *Cdk4, Cdk6, Ccnd1, Ccnd2, p27^*kip1*^, Cdc6, Mcm3, Mcm5, Mcm6, Cdca2* and* Cdca7*, with many of them being repressed by Pax6 (Sansom et al., 2009; Mi et al., 2013a,b). These findings are in line with the notion that Pax6 mainly acts as an important regulator of progenitor cell proliferation during early cortical development as reviewed above. Among the cell cycle genes whose expression is regulated by Pax6, Pax6 direct targets identified to date include *Cdk4*, *Cdk6*, *Mcm3*, *Cdca2*, *Cdca7,* and p27^*kip1*^ (**Table 1**; Duparc et al., 2007; Sansom et al., 2009; Mi et al., 2013a,b).

**Table 1:** Genes that have been identified as direct Pax6 targets in the mouse developing cortex grouped according to the developmental process that they affect.

Process	Gene	Approach							Reference
		ChIP-chip	ChIP	Microarray	EMSA	Luciferase assay	Transgenic mice	qRT-PCR	
Cell fate specification	*Etv1 (Er81)*	X		X	X		X		Tuoc and Stoykova (2008a), Sansom et al. (2009)
	*Olig2*		X			X			Jang and Goldman (2011)
	*Isl1*	X		X					Sansom et al. (2009), Xie et al. (2013)
	*Foxd1*	X		X					Sansom et al. (2009)
Basal progenitor genesis	*Neurod1*	X		X					Sansom et al. (2009)
	*Gadd45g*	X		X					Sansom et al. (2009)
	*Tbr2*	X	X	X				X	Sansom et al. (2009), Tuoc et al. (2013a)
Neurogenesis	*Ngn2*	X		X	X		X		Scardigli et al. (2003), Sansom et al. (2009)
	*Sox4*	X		X					Sansom et al. (2009)
	*Sox11*		X	X					Ninkovic et al. (2013)
	*Pou3f4*		X	X					Ninkovic et al. (2013)
	*Nfib*		X	X					Ninkovic et al. (2013)
	*Cux1*		X		X			X	Tuoc et al. (2013a)
	*Tle1*		X		X			X	Tuoc et al. (2013a)
Cell cycle	*Cdk6*		X		X	X			Mi et al. (2013a)
	*Cdk4*	X		X					Sansom et al. (2009)
	*Mcm3*	X		X					Sansom et al. (2009)
	*Cdca2*	X		X					Sansom et al. (2009)
	*Cdca7*	X		X					Sansom et al. (2009)
	*P27^*kip1*^*		X					X	Duparc et al. (2007)
Cell proliferation	*Pten*	X		X					Sansom et al. (2009)
	*Cdh1*	X		X					Sansom et al. (2009)
	*Lix1*	X		X					Sansom et al. (2009)
	*Hmga2*	X		X					Sansom et al. (2009)
	*Fgfr1*	X		X					Sansom et al. (2009)
Axonal transport	*Kif1b*	X		X		X			Xie et al. (2013)
Unknown	*Snca*	X		X					Xie et al. (2013)

It is interesting to note that in addition to its repressive effect, Pax6 can also activate expression of some cell cycle genes (Holm et al., 2007; Osumi et al., 2008; Sansom et al., 2009; Mi et al., 2013a,b; Xie et al., 2013). For example, Pax6 directly promotes the expression of *Cdk4* in neural stem cells in early cortical development (Sansom et al., 2009). This supports the concept that Pax6 has dual roles to both promote and inhibit cell proliferation in the developing cortex, possibly through the divergent functions of its DNA-binding subdomains (see below; Walcher et al., 2013). Using transcriptome analysis of the cortex in embryos with either gain and loss of Pax6 function, Sansom et al. (2009) showed that Pax6 regulates a core transcriptional network that controls cortical neural stem cell proliferation in a dosage-dependent manner. They found that under normal conditions *in vivo*, Pax6 has the potential to both promote and inhibit stem cell proliferation by exhibiting opposing effects on the expression of different sets of cell cycle genes, but when Pax6 levels are increased, as in transgenic cortex overexpressing Pax6, the neurogenic functions of Pax6 are dominant over its ability to promote proliferation. This study is in line with our finding that at the onset of corticogenesis, the repressive effect of Pax6 on progenitor cell proliferation and the Cdk/Cyclin mediated phosphorylation of pRb is localized to regions of cortex where Pax6 expression is normally highest, indicating a relationship between the level of Pax6 expression and its anti-proliferative effect (Mi et al., 2013a). Given that Pax6 is expressed in a gradient during early cortical development, the dosage-dependent effects of Pax6 on cell proliferation are important in the context of understanding how the cerebral cortex becomes divided into regions with specific cytoarchitectures and functions.

## Pax6 is an Intrinsic Determinant of Neuronal Fate in the Developing Cortex

The observations that Pax6 is specifically expressed by radial glial progenitor cells in the developing cortex and is required for them to acquire their normal morphology, gene expression pattern and cell cycle characteristics (Gotz et al., 1998), suggested that Pax6 may control their ability to generate neurons. To address this question, Heins et al. (2002) used a combination of loss and gain of function approaches. They found that cultured RGCs isolated from the developing cortex of *Pax6^-/-^* mutant mouse embryos generated half as many pure neuronal clones as RGCs from control embryos. Using a tau-GFP transgene to label and quantify neurons by fluorescence activated cell sorting (FACS), they showed that the number of neurons in the cortex of *Pax6^-/-^* mutant mouse embryos was reduced by half at E14.5 and by a third at E16.5 compared to controls, consistent with the previously reported thinner CP in *Pax6^-/-^* cortex (Schmahl et al., 1993). Forced expression of Pax6 in cells dissociated from E14 cortex or in astrocytes from postnatal cortex, via transduction of a retroviral vector containing full length Pax6 cDNA, resulted in a significant increase in the number of pure neuronal clones generated, demonstrating that Pax6 is an intrinsic determinant of neuronal fate in murine cortical progenitors (Heins et al., 2002). Mo and Zecevic (2008) showed that siRNA-mediated knockdown of Pax6 expression in cultured human fetal RGCs led to a significant decrease in the number of neurons produced, suggesting that the role of Pax6 in cortical neurogenesis is maintained from rodents to humans (Mo and Zecevic, 2008).

## Pax6 Confers both Regional and Neurotransmitter Fates

The mammalian CP comprises mainly glutamatergic neurons born in the cortical VZ and GABAergic interneurons (INs). In mouse, most INs are born in the medial ganglionic eminence (MGE; ∼70%) with others coming from the caudal ganglionic eminence (CGE) and areas including the preoptic area (POA; Anderson et al., 2001; Wonders and Anderson, 2006; Fogarty et al., 2007; Gelman et al., 2009; Miyoshi et al., 2010). INs born in the LGE populate the olfactory bulb (OB) and the ventral telencephalon. Most cortical glutamatergic neurons and GABAergic interneurons are generated between E12.5 and E16.5. By E18.5, GABAergic INs are distributed throughout the cortex of wild type embryos. In contrast, the cortex of *Pax6^-/-^* mutant embryos contains large subpial ectopias composed of GABAergic INs (Kroll and O’Leary, 2005). These ectopias develop from E15.5 onward and express markers of LGE-derived INs, including Sp8, rather than markers of MGE-born INs, such as Lhx6. Their development parallels a progressive ventralization of the dorsal telencephalic VZ in *Pax6^-/-^* mutant embryos. At the start of neurogenesis (E12.5–E13.5) the most ventrolateral part of the *Pax6^-/-^* mutant cortex ectopically expresses the ventral telencephalic markers Ascl1 and Gsh2. As neurogenesis progresses ventral telencephalic identities extend dorsally such that by E14.5 most of the dorsal telencephalic VZ exhibits ventral characteristics. This progressive ventralization is accompanied by a dorsal shift in expression of markers of GABAergic INs such as Dlx1/2 and GAD67. Importantly, the INs that form the ectopias are derived from Emx1-expressing cortical progenitors which normally generate glutamatergic neurons, demonstrating that in *Pax6^-/-^* mutant mice, cortical progenitors are re-specified to generate GABAergic INs instead of glutamatergic neurons (Kroll and O’Leary, 2005).

Dlx1/2, Ascl1, and Gsh2 have been shown to promote GABAergic fate, such as the expression of Gad67 (Long et al., 2009a,b; Wang et al., 2013). Thus it is possible that Pax6 normally represses GABAergic identities in the cortex by blocking the expression of these TFs.

In *Pax6^-/-^* embryos, early born CP neurons are correctly specified as dorsal telencephalic neurons, expressing dorsal markers Math2 and Tbr1 (Schuurmans et al., 2004). They fail, however, to adopt a correct glutamatergic phenotype. Interestingly, these early born neurons are not GABAergic either (Schuurmans et al., 2004). Thus, during early corticogenesis, Pax6 participates in the specification of a correct glutamatergic phenotype but is not required to suppress GABAergic identities. Schuurmans et al. (2004) analyzed double mutants for Ngn1 and Ngn2, and showed that, during early stages of corticogenesis (E13.5), neurogenins are required to specify a glutamatergic, cortical phenotype, and repress GABAergic identities. Conversely, cortical neurons born at later stages of corticogenesis (E15.5) acquire a normal cortical and glutamatergic phenotype in *Ngn1^-/-^; Ngn2^-/-^* double mutants whereas in *Pax6^-/-^* embryos and in mutants lacking the orphan nuclear receptor Tlx, they express reduced levels of cortical and glutamatergic markers, compared to controls, and instead acquire a subcortical, GABAergic phenotype (Schuurmans et al., 2004). This study shows that distinct genetic programs operate during early and late corticogenesis to specify cortical regional identity and neurotransmitter fate: neurogenins are required during early stages while Pax6 and Tlx are required during later stages of corticogenesis (Schuurmans et al., 2004).

## Pax6 Confers Laminar Fate

Throughout corticogenesis, newborn neurons migrate to the CP in an inside-out sequence such that deep layers are formed first and superficial layers form later. The final laminar position and subtype of cortical projection neurons is dependent on the time at which progenitors exit the cell cycle. In *Pax6^-/-^* mutant embryos, the SVZ and superficial cortical layers are defective, as shown by absence of expression of Svet1 (which marks the SVZ and superficial layers), while the VZ and deep CP layers are unaffected, as shown by the expression of Otx1 (Tarabykin et al., 2001).

Examination of cortical lamination in *Ngn1^-/-^*; *Ngn2^-/-^* mutants showed that the expression of markers of deep cortical layers is reduced (Tbr1, Er81) while markers of superficial layers are expressed normally, indicating that late-born cortical neurons are correctly specified (Schuurmans et al., 2004). In contrast, no defects are detected in the deeper cortical layers of *Pax6^-/-^*, *Tlx^-/-^*, or *Pax6^-/-^*;* Tlx^-/-^* double mutants. Markers of upper layers, however, are downregulated in Pax6 and Tlx single mutants and completely lost in *Pax6^-/-^*;*Tlx^-/-^* embryos suggesting that Pax6 and Tlx cooperate to specify the identity of late-born cortical neurons (Schuurmans et al., 2004).

Study of cortical lamination in *Pax6^-/-^* mutants is limited by the fact that they die perinatally while cortical neurons do not reach their final location until around postnatal day 8 (P8). Therefore, more recent studies on the role of Pax6 in cortical lamination have used conditional mutants in which *Pax6* is inactivated specifically in the cortex and at later stages of cortical development and which survive postnatally. When *Pax6* was deleted specifically in the cortex from the onset of corticogenesis, markers of superficial layers were either downregulated or lost completely (Tuoc et al., 2009). In contrast, when *Pax6* was deleted in RGCs after the generation of deep cortical layers (between E14.5 and E16.5) using an hGFAP-cre transgenic line, the specification and number of late born neurons was unaffected (Tuoc et al., 2009), leading to the conclusion that Pax6 is not required for the specification of late born cortical neurons and that the defects of superficial layers observed in *Pax6^-/-^* and *Pax6*cKO mutants are a result of earlier defects in RGC proliferation. In a more recent study, Georgala et al. (2011b) used an *Emx1-creErT2* transgenic line to remove Pax6 specifically in the cortex between E12.5 and E13.5, before superficial layer neurons are born. The resulting cKO mutant brains showed a dramatic reduction in the thickness of the superficial cortical layers, compared to control embryos. Late-born neurons were birth-dated by injecting BrdU at E15.5 and stained for expression of Cux1, a marker of upper layer (II to IV) pyramidal neurons. There was a substantial reduction in the number of E15.5 born neurons in the superficial cortical layers of the *Pax6-cKO* mutants and many fewer late-born neurons expressed Cux1 (Georgala et al., 2011b), indicating that Pax6 plays a crucial role in generation and specification of late born cortical neurons. Note that Pax6 protein was lost slightly earlier in the conditional mutants generated by Georgala et al. (2011b) than in those used by Tuoc et al. (2009), potentially explaining the difference in outcomes between the two studies.

## Direct Transcriptional Targets of Pax6 Regulate Basal Progenitor Genesis, Neurogenesis, and Cell Fate Specification

Pax6 exerts many of its effects, at least in part, by directly regulating the expression of specific target genes (**Table 1**). As described above, work in the mouse has shown that Pax6 expressing APs that give rise to BPs transiently express the proneural TF Neurogenin 2 (Ngn2). BPs do not express Pax6 but do express the TF Tbr2. Loss of Pax6 function results in loss of Ngn2 expression in the cortical VZ and downregulation of Tbr2 in the SVZ (Stoykova et al., 2000; Toresson et al., 2000; Yun et al., 2001; Scardigli et al., 2003; Holm et al., 2007; Quinn et al., 2007; Sansom and Livesey, 2009), suggesting that the development of BPs is impaired. Conversely, Pax6 overexpression results in a marked increase in both Ngn2 and Tbr2 expression (Scardigli et al., 2003; Sansom and Livesey, 2009), each of which have been shown to be directly regulated by Pax6 (Scardigli et al., 2003; Sansom and Livesey, 2009; Tuoc et al., 2013a). Scardigli et al. (2003) showed that Pax6 directly binds and regulates the activity of the E1 enhancer of *Ngn2*, which regulates *Ngn2* expression in the cortex and the ventral spinal cord.

Another direct target that is positively regulated by Pax6 is the anti-neurogenic transcriptional co-repressor Tle1, a member of the Groucho/transducin-like enhancer of split (Gro/Tle) family (Sansom et al., 2009; Tuoc et al., 2013a). Gro/Tle proteins are expressed in proliferating neural progenitor cells where they promote maintenance of the undifferentiated state by inhibiting/delaying neuronal differentiation (Buscarlet and Stifani, 2007; Jennings and Ish-Horowicz, 2008).

Pax6 also directly inhibits the expression of Olig2 (Jang and Goldman, 2011), a TF critical for glial cell fate determination (Marshall et al., 2005). Olig2 is expressed in a subset of cells in the rat neonatal SVZ, which differentiate into glia. Forced expression of Pax6 in these Olig2+ cells, using a retrovirus, caused downregulation of Olig2 and a shift toward neuronal fate (Jang and Goldman, 2011). These authors showed that Pax6 can directly bind to Olig2’s promoter and repress its activity.

Pax6 directly regulates markers of specific neuronal subtypes including Er81 and Cux1 (Tuoc and Stoykova, 2008a; Sansom et al., 2009; Tuoc et al., 2013a). Er81 is a TF expressed in cortical progenitors, in a rostrolateral-high to caudomedial-low gradient, and in a subset of pyramidal cells scattered through layer 5 (Yoneshima et al., 2006; Tuoc and Stoykova, 2008a). In the absence of Pax6, cortical progenitors do not express Er81 and the rostrolateral cortex lacks Er81 positive layer 5 neurons. Cux1 is a marker of upper layer (II to IV) pyramidal neurons and functions as a negative regulator of dendritic complexity for these neurons (Cubelos et al., 2010, 2014; Li et al., 2010). As mentioned above, loss of Pax6 results in a dramatic reduction of Cux1+ upper layer neurons, suggesting that the specification of late born neurons is impaired (Georgala et al., 2011b).

How does a single gene exert such a wide range of biological effects? Part of the answer to this question is suggested by the findings that the Pax6 protein contains two distinct DNA-binding domains, one of which is regulated by alternate splicing and that Pax6 is able to interact with a number of co-factors, which influence its activity. This issue is discussed in the next section.

## Complexity of Pax6 DNA-Binding Properties and its Distinct Functions

**FIGURE 7:**
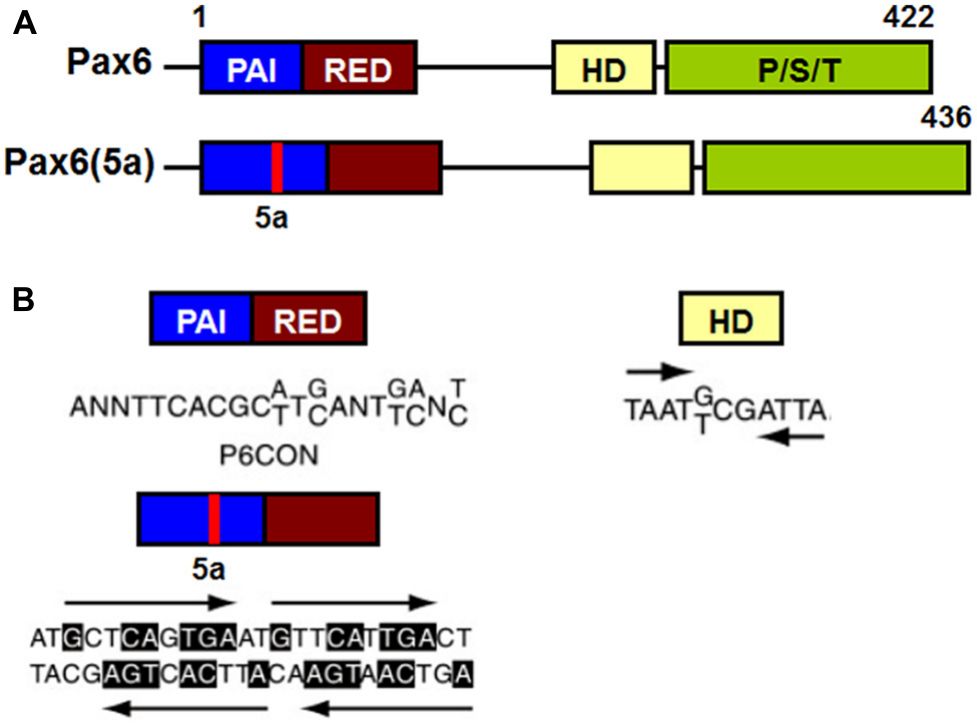
Schematic representation of functional domains in Pax6 and Pax6(5a) and their “optimal” DNA binding sites. **(A)** Pax6 and Pax6(5a) differ in a 14 amino acid insertion in the PAI subdomain. **(B)** “Optimal” DNA-binding sites for Pax6 PD, PD5a and HD. P6CON and 5aCON sequences were generated by selection procedure (Epstein et al., 1994). P6CON is recognized by monomeric PD, while 5aCON contains four binding sites for PD5a that can bind up to four PD5a units. The “optimal” binding site of Pax6 HD is a dimer with typical HD binding sequences (ATTA/TAAT) separated by three nucleotides (G/T)CG (Czerny and Busslinger, 1995).

Pax6 binds to target DNA sequences through one or both of two DNA-binding domains, the paired domain (PD) and the homeodomain (HD; Bertuccioli et al., 1996; Jun and Desplan, 1996; Sheng et al., 1997; Singh et al., 2000). Transcriptional regulation of Pax6 target genes is mediated by a carboxy terminal proline/serine/threonine (PST) rich transactivation domain (Singh et al., 1998, 2001; Tang et al., 1998; **Figure 7**).

The PD is an evolutionarily conserved 128 amino acid DNA-binding domain shared by all members of the paired box (Pax) family of TFs (Pax1-Pax9; Chalepakis et al., 1992; Gruss and Walther, 1992). The Pax6 PD can be structurally and functionally separated into two independent DNA-binding globular helix-turn-helix subdomains, PAI and RED (Puschel et al., 1992; Czerny et al., 1993; Jun and Desplan, 1996; **Figure 7A**). The well-established DNA-binding motif for canonical Pax6 PD, known as P6CON, is a bipartite 20 base pair DNA sequence whose 5^′^-half is recognized by the PAI subdomain and 3^′^-half by the RED subdomain (Czerny et al., 1993; Epstein et al., 1994; **Figure 7A**). The crystal structure of the Pax6 PD – P6CON complex revealed direct interactions between both PAI and RED subdomains, the linker regions of PAI and RED subdomains and the N-terminal β-turn subdomain with individual bases and phosphate residues of cognate DNA (Czerny et al., 1993; Epstein et al., 1994; Xu et al., 1999a). Pax6 also contains an internal, paired-type HD (Czerny et al., 1999; Eberhard and Busslinger, 1999) which binds DNA in the form of a homodimer recognizing a symmetric binding site (Wilson et al., 1993; Eberhard and Busslinger, 1999; Mikkola et al., 2001; **Figure 7B**).

Although different Pax6 DNA-binding domains can independently bind to their “optimal" binding sites, using individual consensus binding motif to predict and confirm candidate Pax6-binding sites has proven to be very difficult (Shimoda et al., 2002; Visel et al., 2007; Wolf et al., 2009). Unlike sequence specific DNA-binding proteins which harbor only one DNA-binding domain, Pax6 exhibits increased DNA-binding complexity because of its multiple DNA-binding subdomains (PAI, RED, and HD) which can be used in varying combinations when binding to DNA (Cvekl et al., 2004; Grapp et al., 2009). A recent study further reinforced the complexity of Pax6 DNA-binding properties by systematically examining coordinated actions of different Pax6 binding subdomains and identified a number of novel variants of Pax6 DNA-binding motifs which can be bound by different combinations of Pax6 binding subdomains (Xie and Cvekl, 2011). As Pax6 can function either as a transcriptional activator or repressor (Wolf et al., 2009), it is possible that some of these binding motif variants may induce structural changes in Pax6 proteins that enable them to switch their function between transcriptional activation and repression.

The complexity of the DNA binding properties of different Pax6 subdomains implies that the subregions exert distinct functions during embryonic cortical development. Pax6 mutant mouse lines with disruption of either the PD or the HD have been used to investigate the impact of different Pax6 binding domains on telencephalic development. Haubst et al. (2004) showed that the PD alone is necessary and sufficient for the regulation of most aspects of telencephalic development including neurogenesis, progenitor cell proliferation, and patterning in the developing cortex. In contrast, HD-specific mutations affected only subtle aspects of telencephalic development, such as establishment of the PSPB, and had no obvious effect on neurogenesis or proliferation (Haubst et al., 2004). This suggests that the majority of Pax6’s effects during telencephalic development are mediated by the PD with potentially different contributions by its subdomains. A recent study further dissected the roles of the different subdomains of the Pax6 PD during telencephalic development by examining mice with point mutations in its individual subdomains PAI and RED (Walcher et al., 2013). This showed that both PAI and RED act largely in a redundant manner in patterning of the telencephalon, with only the PAI mutation resulting in the ectopic expression of subpallial genes such as *Gsx2* and *Olig2* into the pallium, suggesting that RED subdomain on its own is not sufficient to regulate telencephalic patterning. In addition, neurogenesis was affected only by the PAI subdomain mutation, phenocopying the neurogenic defects observed in full *Pax6^-/-^* mutants. Strikingly, this study also revealed that subdomains of Pax6 PD have distinct roles in regulating cortical progenitor cell proliferation, with mutations affecting the PAI and RED subdomains respectively reducing and increasing the number of mitoses.

## Alternatively Spliced Isoforms of Pax6 and their Functions

In addition to Pax6 and Pax6(5a) a number of other splice variants of Pax6 have been reported, named p43, p33 and p32, each of which is less abundant and mostly localized in the cytoplasm (Carriere et al., 1993; Jaworski et al., 1997; Pinson et al., 2006). The 14 amino acid insertion in Pax6(5a) destroys the DNA-binding capacity of the PAI subdomain, leaving the RED subdomain of the PD and the intact HD for DNA binding (Epstein et al., 1994; Kozmik et al., 1997). The PD in Pax6(5a) binds to a distinct DNA sequence from that bound by the canonical Pax6 PD, that has been named 5aCON (Epstein et al., 1994). Direct comparison of the DNA binding sites recognized by Pax6 PD and PD(5a) showed partial sequence homology between the 3’ halves of P6CON and 5aCON (Epstein et al., 1994; Duncan et al., 1998, 2000). This homology allows Pax6 (which contains the canonical PD) to bind to both P6CON and 5aCON sites, whereas Pax6(5a) can only interact with the 5aCON site (Epstein et al., 1994).

The majority of published molecular studies have focused on Pax6, as it contains the canonical PD and appears to be more abundant than Pax6(5a) (Carriere et al., 1993; Richardson et al., 1995; Koroma et al., 1997). During early development, *Pax6* and *Pax6(5a)* transcripts are expressed in a ratio of 8:1 in the mouse forebrain, falling to 3:1 between E12.5 and E14.5 (Kozmik et al., 1997; Pinson et al., 2006). A reduced ratio between Pax6 and Pax6(5a) was also seen during chick retina development (Azuma et al., 2005). *PAX6* and *PAX6(5a)* transcripts are equally abundant in the human adult lens epithelium and cornea, as well as in monkey retina (Zhang et al., 2001). These findings raise the possibility that expression of the two main Pax6 isoforms are regulated in a tissue- and temporal-specific manner, thereby affecting which target genes they recognize.

Although Pax6 and Pax6(5a) are commonly co-expressed, there is evidence that they exert distinct functions during embryonic development. For example, overexpression of either Pax6 or Pax6(5a) in chick retina showed that both isoforms led to increased retinal progenitor cell proliferation, but Pax6(5a) induced ectopic differentiation of the retina to a stronger degree than Pax6 (Azuma et al., 2005). Similarly, Pax6(5a) strongly induces neural differentiation of murine embryonic stem cells *in vitro*, while Pax6 does not have such a strong effect (Shimizu et al., 2009). In the embryonic mouse forebrain, the different roles of the two Pax6 isoforms in regulation of cell proliferation and differentiation were also established from studies of Pax6 mutants. It was shown that mutations that abolish the binding property of the PD affect both proliferation and differentiation, while a PD(5a) mutation only affects proliferation and has no effect on cell fate/differentiation in mouse developing telencephalon (Haubst et al., 2004). In a more recent study, Pax6(5a) specific target genes were identified through microarray analyses of gene expression profiles in cell lines stably expressing either Pax6 or Pax6(5a) (Kiselev et al., 2012). This revealed that a number of genes involved in cell proliferation, differentiation, and migration are differentially regulated by Pax6 and Pax6(5a), providing a potential molecular basis by which Pax6 and Pax6(5a) could exert their distinct biological functions.

## Co-Factors Influence Pax6’s Direct Regulation of Target Genes Involved in Embryonic and Adult Neurogenesis

It is becoming clear that transcriptional regulation is highly dependent on the molecular and cellular context, one reason for which is that TFs do not act on their own but are dependent on interactions with other co-factors to exert their functions (Oikawa and Yamada, 2003; Westerman et al., 2003). These interactions introduce more specificity into the regulatory function of a given TF in a particular cellular context. A number co-factors that can interact with Pax6 have been identified. For example, Pax6 cooperates with Sox2 to target the lens-specific enhancer of the δ*-crystallin* gene (Kamachi et al., 2001); with MDIA to influence Pax6’s functions in cerebellar granule cells (Tominaga et al., 2002); with Trim11 to mediate Pax6 degradation through the ubiquitin proteasome system (UPS) and modulate Pax6 transcriptional activity (Cooper and Hanson, 2005; Tuoc and Stoykova, 2008b). More recently, Brg1/Brm associated factors complex (BAF) and Meis2 have been identified as two new co-factors that affect Pax6’s direct regulation of target genes expression, in turn regulating its role in both embryonic and adult neurogenesis (Ninkovic et al., 2013; Tuoc et al., 2013a,b; Agoston et al., 2014).

## BAF Complex

**FIGURE 8:**
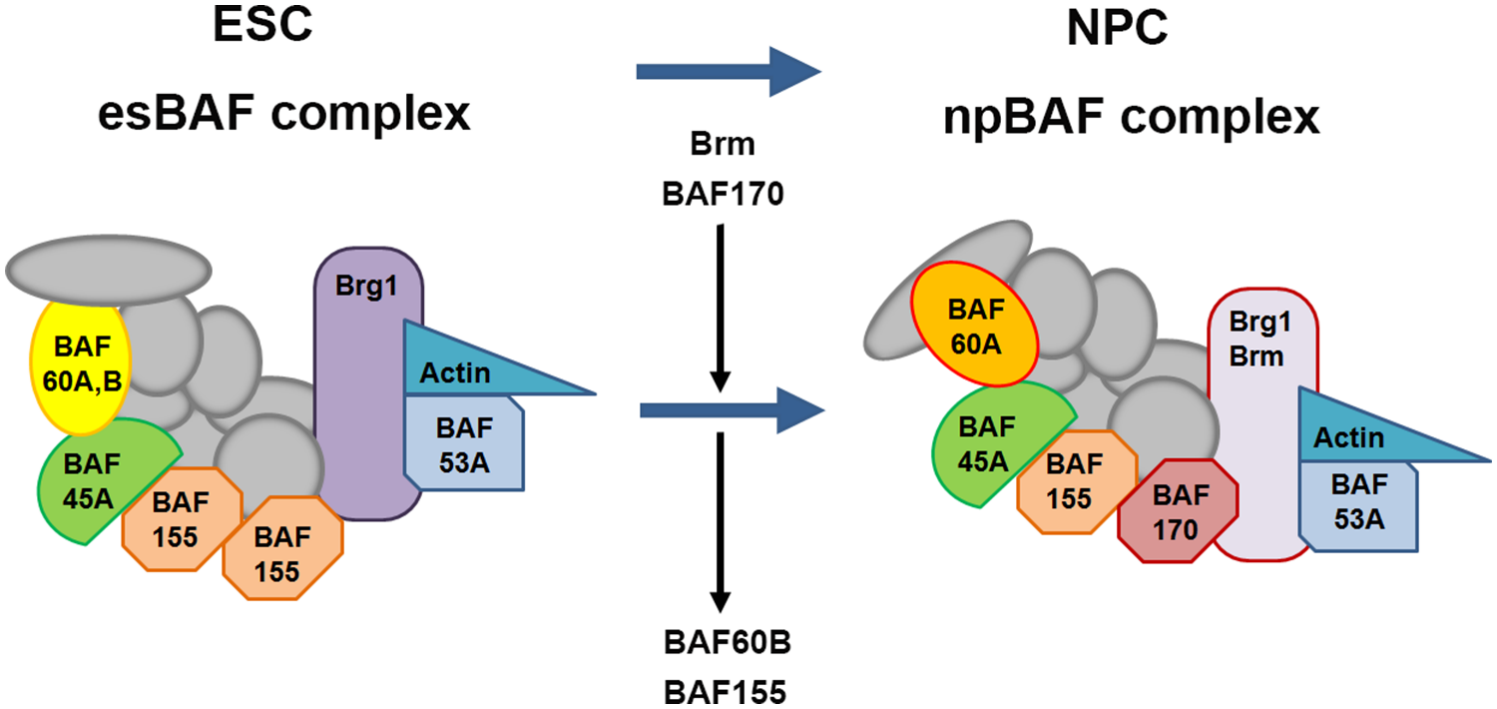
Changes in subunit composition of BAF complexes during differentiation from ES cells to neural progenitor cells. BAF complexes undergo progressive changes in subunit composition during the transition from embryonic stem cells (ESCs) to neural progenitor cells (NPCs). The BAF complex in ESCs is called esBAF; in neuronal progenitors, npBAF. As ESCs differentiate into NPCs, The esBAF complexes incorporate Brm and BAF170, while excludes BAF60B and BAF155, thereby forming the neural progenitor-specific BAF (npBAF) complexes in NPCs.

A number of chromatin remodeling factors are known to play an important role during mammalian neural development, including the BAF complex (Ho et al., 2009a,b; Wu, 2012; Yip et al., 2012; Tuoc et al., 2013a,b). Interestingly, the function of the BAF complex in regulating neural development is dependent on its subunit composition. In mammals, BAF complexes contain two ATPase subunits Brg1 or Brm (Brahma), which are mutually exclusive and essential for remodeling activity, in combination with at least 15 different BAF (Brg1/Brm-associated factor) subunits (Ho et al., 2009a; Singhal et al., 2010). Certain BAF subunits have restricted expression patterns and thus could define tissue- or cell-type-specific BAF complexes. For example, the composition of BAF complexes in ES cells (esBAF) is defined by the incorporation of Brg1 but not Brm, BAF155 but not BAF170, and BAF60A but not BAF60C (**Figure 8**; Ho et al., 2009a,b). However, during the transition from ES cells to neural progenitors, the composition of BAF complexes is correspondingly changed, defining the neural progenitor specific BAF complex (npBAF; Lessard et al., 2007; Yan et al., 2008; Ho et al., 2009a). This process is accompanied by induction of BAF170 expression, replacing one of the BAF155 subunits and the recruitment of Brm as the ATP-ase subunit in the npBAF complex.

In two recent studies, Tuoc et al. (2013a,b), described an elegant model in which time-specific binding of BAF subunits including BAF170, BAF155, and Brm-ATPase to Pax6 modulates the expression of Pax6 target genes (*Tbr2, Cux1,* and *Tle1*) involved in the specification and generation of BPs and upper layer neuronal fate in the developing cortex. Interestingly, this model involves competition between BAF170 and BAF155 subunits in the BAF complex, which modulates chromatin modifications and binding of the Pax6/REST co-repressor complex to Pax6 target genes, thereby affecting their expression. During early neurogenesis (E12.5 to E14.5), the presence of BAF170 in the Pax6-BAF complex prevents the euchromatin state (a lightly packed form of chromatin) of the Pax6 target genes *Tbr2, Cux1* and *Tle1*, limiting the ability of Pax6 to bind them, thereby inhibiting their expression and promoting direct neurogenesis (RGCs directly give rise to neurons) at this developmental stage. In contrast, after E14.5 the expression of BAF170 decreases, accompanied by enhanced expression of BAF155, leading to a relaxed chromatin state of the promoters of the Pax6 target genes mentioned above, thereby enhancing their expression and in turn promoting indirect neurogenesis (RGCs indirectly give rise to neurons through generating BPs). This study provides novel insight into the interaction between BAF complex and Pax6 in the generation of BPs and the mode of neurogenesis in the developing cortex. In another recent study, Ninkovic et al. (2013) showed that Pax6 directly interacts with Brg1-ATPase of the BAF complex to modulate expression of genes involved in neuronal fate specification in adult neural progenitors. This study indicated that direct interaction of Pax6 with the Brg1/BAF complex enhances the expression of Pax6 target neurogenic TFs including *Sox11, Nfib,* and *Pou3f4* which form a cross-regulatory network driving neurogenic fate during adult neurogenesis. This study improved our understanding of Pax6-mediated adult neurogenesis and shed light on molecular mechanisms underlying the function of Pax6 in cell fate specification and conversion.

## Meis2

Meis2 belongs to the TALE (three amino acid loop extension) family of atypical HD-containing TFs. It can form heteromeric complexes with other transcriptional regulators to exert its functions during a range of biological processes (Moskow et al., 1995; Chang et al., 1997; Knoepfler et al., 1999; Moens and Selleri, 2006). Meis proteins control cell cycle progression and cell fate specification of different stem and progenitor cell types in a variety of tissues including liver, heart, retina, and brain during development (Mercader et al., 1999; Hisa et al., 2004; Bessa et al., 2008; Heine et al., 2008; Agoston and Schulte, 2009; Choe et al., 2009; Agoston et al., 2012, 2014; Paige et al., 2012). Recently the functional importance of Meis2 in adult neurogenesis has been discovered. It is involved in coordinated actions with Pax6 (Agoston et al., 2014). During mouse adult neurogenesis, Meis2 is strongly expressed in the SVZ and rostral migratory stream (RMS) and in a subset of OB interneurons (Agoston et al., 2014). Agoston et al. (2014) found that Meis2 can physically interact with Pax6 in cells in the SVZ and OB. This study also showed that the pro-neurogenic function of Pax6 in the adult SVZ requires direct interaction with Meis2. Meis2 has transcriptional activator function and Agoston et al. (2014) further confirmed that it acts as a Pax6 co-factor, binding to regulatory elements of the *Dcx* gene to promote its expression during adult SVZ neurogenesis.

## Conclusion

The TF Pax6 is one of the most intensively studied high-level developmental regulators, playing crucial roles in brain development and implicated in disease. Numerous studies, including those from our group, have shown that in both humans and mice many abnormalities resulting from mutations in *PAX6/Pax6* are in the cerebral cortex, where they arise largely because Pax6 is required for spatio-temporal control of many biological processes including cortical stem and progenitor cell proliferation and differentiation, neurogenesis, cell and laminar fate specification, neuronal migration, axon guidance and cortical arealization during early corticogenesis. However, we are a long way from having a comprehensive knowledge of exactly how Pax6 exerts such complex actions. In particular, there are at least three major challenges toward improved understanding of Pax6 functions. First, how does such highly complex expression pattern of Pax6 come about? As described above, the transcriptional regulation of *Pax6* is particularly complex and involves both short-range and long-range control mechanisms. Future studies are required to gain better knowledge of tissue- and time-specific regulatory elements responsible for spatiotemporally and quantitatively correct expression of Pax6. Secondly, how does Pax6 expression level influence its functional outputs? Our studies along with others have shown that Pax6 expression levels are crucial for its functions such as regulating progenitor cell proliferation and differentiation, and that mutations affecting Pax6 protein levels cause neurodevelopmental disorders in both humans and mice. Now it is time to ask exactly how Pax6 levels influence its actions at the molecular level. It will be of great interest to examine how varying levels of Pax6 influence its molecular actions at different cell cycle phases, which will be critical to obtain a clearer understanding of how Pax6 functions during the dynamics of cell division. Finally and probably most importantly, we are still far away from a comprehensive understanding of the complex gene networks that Pax6 regulates and how these networks change spatially and temporally during the course of cortical development. This represents a major challenge since Pax6 likely exerts direct and indirect regulation of different sets of downstream genes simultaneously, with context-dependent variation in the strength of their positive or negative effects.

## Conflict of Interest Statement

The authors declare that the research was conducted in the absence of any commercial or financial relationships that could be construed as a potential conflict of interest.

## References

[B1] Agoston Z. Heine P. Brill M. S. Grebbin B. M. Hau A. C. Kallenborn-Gerhardt W. (2014). Meis2 is a Pax6 co-factor in neurogenesis and dopaminergic periglomerular fate specification in the adult olfactory bulb. *Development* 141 28–38 10.1242/dev.097295 24284204

[B2] Agoston Z. Li N. Haslinger A. Wizenmann A. Schulte D. (2012). Genetic and physical interaction of Meis2 Pax3 and Pax7 during dorsal midbrain development. *BMC Dev. Biol.* 12:10 10.1186/1471-213X-12-10 PMC3313853 22390724

[B3] Agoston Z. Schulte D. (2009). Meis2 competes with the Groucho co-repressor Tle4 for binding to Otx2 and specifies tectal fate without induction of a secondary midbrain-hindbrain boundary organizer. *Development* 136 3311–3322 10.1242/dev.037770 19736326

[B4] Anderson S. A. Marin O. Horn C. Jennings K. Rubenstein J. L. (2001). Distinct cortical migrations from the medial and lateral ganglionic eminences. *Development* 128 353–363. 11152634 10.1242/dev.128.3.353

[B5] Angevine J. B. Jr. Sidman R. L. (1961). Autoradiographic study of cell migration during histogenesis of cerebral cortex in the mouse. *Nature* 192 766–768 10.1038/192766b0 17533671

[B6] Aota S. Nakajima N. Sakamoto R. Watanabe S. Ibaraki N. Okazaki K. (2003). Pax6 autoregulation mediated by direct interaction of Pax6 protein with the head surface ectoderm-specific enhancer of the mouse Pax6 gene. *Dev. Biol.* 257 1–13 10.1016/S0012-1606(03)00058-7 12710953

[B7] Arai Y. Pulvers J. N. Haffner C. Schilling B. Nüsslein I. Calegari F. (2011). Neural stem and progenitor cells shorten S-phase on commitment to neuron production. *Nat. Commun.* 2 154 10.1038/ncomms1155 PMC3105305 21224845

[B8] Azuma N. Tadokoro K. Asaka A. Yamada M. Yamaguchi Y. Handa H. (2005). The Pax6 isoform bearing an alternative spliced exon promotes the development of the neural retinal structure. *Hum. Mol. Genet.* 14 735–745 10.1093/hmg/ddi069 15677484

[B9] Bamiou D. E. Campbell N. G. Musiek F. E. Taylor R. Chong W. K. Moore A. (2007a). Auditory and verbal working memory deficits in a child with congenital aniridia due to a PAX6 mutation. *Int. J. Audiol.* 46 196–202 10.1080/14992020601175952 17454233

[B10] Bamiou D. E. Free S. L. Sisodiya S. M. Chong W. K. Musiek F. Williamson K. A. (2007b). Auditory interhemispheric transfer deficits, hearing difficulties, and brain magnetic resonance imaging abnormalities in children with congenital aniridia due to PAX6 mutations. *Arch. Pediatr. Adolesc. Med.* 161 463–469 10.1001/archpedi.161.5.463 17485622

[B11] Bamiou D. E. Musiek F. E. Sisodiya S. M. Free S. L. Davies R. A. Moore A. (2004). Deficient auditory interhemispheric transfer in patients with PAX6 mutations. *Ann. Neurol.* 56 503–509 10.1002/ana.20227 15389894

[B12] Beaulieu C. (1993). Numerical data on neocortical neurons in adult rat, with special reference to the GABA population. *Brain Res.* 609 284–292 10.1016/0006-8993(93)90884-P 8508310

[B13] Bel-Vialar S. Medevielle F. Pituello F. (2007). The on/off of Pax6 controls the tempo of neuronal differentiation in the developing spinal cord. *Dev. Biol.* 305 659–673 10.1016/j.ydbio.2007.02.012 17399698

[B14] Bentivoglio M. Mazzarello P. (1999). The history of radial glia. *Brain Res. Bull.* 49 305–315 10.1016/S0361-9230(99)00065-9 10452351

[B15] Berger J. Berger S. Tuoc T. C. D’amelio M. Cecconi F. Gorski J. A. (2007). Conditional activation of Pax6 in the developing cortex of transgenic mice causes progenitor apoptosis. *Development* 134 1311–1322 10.1242/dev.02809 17329367

[B16] Berry M. Rogers A. W. (1965). The migration of neuroblasts in the developing cerebral cortex. *J. Anat.* 99 691–709. 5325778 PMC1270716

[B17] Bertuccioli C. Fasano L. Jun S. Wang S. Sheng G. Desplan C. (1996). In vivo requirement for the paired domain and homeodomain of the paired segmentation gene product. *Development* 122 2673–2685. 8787742 10.1242/dev.122.9.2673

[B18] Bessa J. Tavares M. J. Santos J. Kikuta H. Laplante M. Becker T. S. (2008). meis1 regulates cyclin D1 and c-myc expression, and controls the proliferation of the multipotent cells in the early developing zebrafish eye. *Development* 135 799–803 10.1242/dev.011932dev.011932 18216175

[B19] Betizeau M. Cortay V. Patti D. Pfister S. Gautier E. Bellemin-Menard A. (2013). Precursor diversity and complexity of lineage relationships in the outer subventricular zone of the primate. *Neuron* 80 442–457 10.1016/j.neuron.2013.09.032S0896-6273(13)00863-5 24139044

[B20] Bishop K. M. Goudreau G. O’leary D. D. (2000). Regulation of area identity in the mammalian neocortex by Emx2 and Pax6. *Science* 288 344–349 10.1126/science.288.5464.344 10764649

[B21] Bishop K. M. Rubenstein J. L. O’leary D. D. (2002). Distinct actions of Emx1 Emx2 and Pax6 in regulating the specification of areas in the developing neocortex. *J. Neurosci.* 22 7627–7638. 12196586 10.1523/JNEUROSCI.22-17-07627.2002 PMC6757966

[B22] Britz O. Mattar P. Nguyen L. Langevin L. M. Zimmer C. Alam S. (2006). A role for proneural genes in the maturation of cortical progenitor cells. *Cereb. Cortex* 16(Suppl. 1) i138–i151 10.1093/cercor/bhj168 16766700

[B23] Buscarlet M. Stifani S. (2007). The ‘Marx’ of Groucho on development and disease. *Trends Cell Biol.* 17 353–361 10.1016/j.tcb.2007.07.002 17643306

[B24] Cameron R. S. Rakic P. (1991). Glial cell lineage in the cerebral cortex: a review and synthesis. *Glia* 4 124–137 10.1002/glia.440040204 1827774

[B25] Caric D. Gooday D. Hill R. E. Mcconnell S. K. Price D. J. (1997). Determination of the migratory capacity of embryonic cortical cells lacking the transcription factor Pax-6. *Development* 124 5087–5096. 9362466 10.1242/dev.124.24.5087

[B26] Carriere C. Plaza S. Martin P. Quatannens B. Bailly M. Stehelin D. (1993). Characterization of quail Pax-6 (Pax-QNR) proteins expressed in the neuroretina. *Mol. Cell. Biol.* 13 7257–7266 10.1128/MCB.13.12.7257 8246948 PMC364796

[B27] Cartier L. Laforge T. Feki A. Arnaudeau S. Dubois-Dauphin M. Krause K. H. (2006). Pax6-induced alteration of cell fate: shape changes, expression of neuronal alpha tubulin, postmitotic phenotype, and cell migration. *J. Neurobiol.* 66 421–436 10.1002/neu.20225 16425216

[B28] Chalepakis G. Tremblay P. Gruss P. (1992). Pax genes, mutants and molecular function. *J. Cell Sci. Suppl*. 16 61–67 10.1242/jcs.1992.Supplement_16.8 1363663

[B29] Chang C. P. Jacobs Y. Nakamura T. Jenkins N. A. Copeland N. G. Cleary M. L. (1997). Meis proteins are major in vivo DNA binding partners for wild-type but not chimeric Pbx proteins. *Mol. Cell. Biol.* 17 5679–5687. 9315626 10.1128/mcb.17.10.5679 PMC232416

[B30] Choe S. K. Lu P. Nakamura M. Lee J. Sagerstrom C. G. (2009). Meis cofactors control HDAC and CBP accessibility at Hox-regulated promoters during zebrafish embryogenesis. *Dev. Cell* 17 561–567 10.1016/j.devcel.2009.08.007 19853569 PMC2768649

[B31] Chow R. L. Altmann C. R. Lang R. A. Hemmati-Brivanlou A. (1999). Pax6 induces ectopic eyes in a vertebrate. *Development* 126 4213–4222. 10477290 10.1242/dev.126.19.4213

[B32] Cooper S. T. Hanson I. M. (2005). A screen for proteins that interact with PAX6: C-terminal mutations disrupt interaction with HOMER3 DNCL1 and TRIM11. *BMC Genet.* 6:43 10.1186/1471-2156-6-43 PMC1208879 16098226

[B33] Crolla J. A. van Heyningen V. (2002). Frequent chromosome aberrations revealed by molecular cytogenetic studies in patients with aniridia. *Am. J. Hum. Genet.* 71 1138–1149 10.1086/344396 12386836 PMC385089

[B34] Cubelos B. Briz C. G. Esteban-Ortega G. M. Nieto M. (2014). Cux1 and Cux2 selectively target basal and apical dendritic compartments of layer II-III cortical neurons. *Dev. Neurobiol.* 75 163–172 10.1002/dneu.22215 25059644

[B35] Cubelos B. Sebastian-Serrano A. Beccari L. Calcagnotto M. E. Cisneros E. Kim S. (2010). Cux1 and Cux2 regulate dendritic branching, spine morphology, and synapses of the upper layer neurons of the cortex. *Neuron* 66 523–535 10.1016/j.neuron.2010.04.038 20510857 PMC2894581

[B36] Cvekl A. Yang Y. Chauhan B. K. Cveklova K. (2004). Regulation of gene expression by Pax6 in ocular cells: a case of tissue-preferred expression of crystallins in lens. *Int. J. Dev. Biol.* 48 829–844 10.1387/ijdb.041866ac 15558475 PMC2080872

[B37] Czerny T. Busslinger M. (1995). DNA-binding and transactivation properties of Pax-6: three amino acids in the paired domain are responsible for the different sequence recognition of Pax-6 and BSAP (Pax-5). *Mol. Cell. Biol.* 15 2858–2871. 7739566 10.1128/mcb.15.5.2858 PMC230517

[B38] Czerny T. Halder G. Kloter U. Souabni A. Gehring W. J. Busslinger M. (1999). twin of eyeless, a second Pax-6 gene of *Drosophila*, acts upstream of eyeless in the control of eye development. *Mol. Cell* 3 297–307 10.1016/S1097-2765(00)80457-8 10198632

[B39] Czerny T. Schaffner G. Busslinger M. (1993). DNA sequence recognition by Pax proteins: bipartite structure of the paired domain and its binding site. *Genes Dev.* 7 2048–2061 10.1101/gad.7.10.2048 8406007

[B40] Davis L. K. Meyer K. J. Rudd D. S. Librant A. L. Epping E. A. Sheffield V. C. (2008). Pax6 3’ deletion results in aniridia, autism and mental retardation. *Hum. Genet.* 123 371–378 10.1007/s00439-008-0484-x 18322702 PMC2719768

[B41] Dehay C. Kennedy H. (2007). Cell-cycle control and cortical development. *Nat. Rev. Neurosci.* 8 438–450 10.1038/nrn2097 17514197

[B42] Dominguez M. Ferres-Marco D. Gutierrez-Avino F. J. Speicher S. A. Beneyto M. (2004). Growth and specification of the eye are controlled independently by Eyegone and Eyeless in *Drosophila melanogaster*. *Nat. Genet.* 36 31–39 10.1038/ng1281 14702038

[B43] Duncan M. K. Cvekl A. Li X. Piatigorsky J. (2000). Truncated forms of Pax-6 disrupt lens morphology in transgenic mice. *Invest. Ophthalmol. Vis. Sci.* 41 464–473. 10670477

[B44] Duncan M. K. Haynes J. I. Cvekl A. Piatigorsky J. (1998). Dual roles for Pax-6: a transcriptional repressor of lens fiber cell-specific beta-crystallin genes. *Mol. Cell. Biol.* 18 5579–5586. 9710641 10.1128/mcb.18.9.5579 PMC109142

[B45] Duparc R. H. Abdouh M. David J. Lepine M. Tetreault N. Bernier G. (2007). Pax6 controls the proliferation rate of neuroepithelial progenitors from the mouse optic vesicle. *Dev. Biol.* 301 374–387 10.1016/j.ydbio.2006.11.006 17157287

[B46] Eberhard D. Busslinger M. (1999). The partial homeodomain of the transcription factor Pax-5 (BSAP) is an interaction motif for the retinoblastoma and TATA-binding proteins. *Cancer Res.* 59(Suppl. 7) 1716s–1724s. 10197586

[B47] Ellison-Wright Z. Heyman I. Frampton I. Rubia K. Chitnis X. Ellison-Wright I. (2004). Heterozygous PAX6 mutation, adult brain structure and fronto-striato-thalamic function in a human family. *Eur. J. Neurosci.* 19 1505–1512 10.1111/j.1460-9568.2004.03236.x 15066147

[B48] Englund C. Fink A. Lau C. Pham D. Daza R. A. Bulfone A. (2005). Pax6 Tbr2 and Tbr1 are expressed sequentially by radial glia, intermediate progenitor cells, and postmitotic neurons in developing neocortex. *J. Neurosci.* 25 247–251 10.1523/JNEUROSCI.2899-04.2005 15634788 PMC6725189

[B49] Epstein J. Cai J. Glaser T. Jepeal L. Maas R. (1994). Identification of a Pax paired domain recognition sequence and evidence for DNA-dependent conformational changes. *J. Biol. Chem.* 269 8355–8361. 8132558

[B50] Estivill-Torrus G. Pearson H. Van Heyningen V. Price D. J. Rashbass P. (2002). Pax6 is required to regulate the cell cycle and the rate of progression from symmetrical to asymmetrical division in mammalian cortical progenitors. *Development* 129 455–466. 11807037 10.1242/dev.129.2.455

[B51] Fantes J. Redeker B. Breen M. Boyle S. Brown J. Fletcher J. (1995). Aniridia-associated cytogenetic rearrangements suggest that a position effect may cause the mutant phenotype. *Hum. Mol. Genet.* 4 415–422 10.1093/hmg/4.3.415 7795596

[B52] Farkas L. M. Huttner W. B. (2008). The cell biology of neural stem and progenitor cells and its significance for their proliferation versus differentiation during mammalian brain development. *Curr. Opin. Cell Biol.* 20 707–715 10.1016/j.ceb.2008.09.008 18930817

[B53] Fietz S. A. Kelava I. Vogt J. Wilsch-Brauninger M. Stenzel D. Fish J. L. (2010). OSVZ progenitors of human and ferret neocortex are epithelial-like and expand by integrin signaling. *Nat. Neurosci.* 13 690–699 10.1038/nn.2553nn.2553 20436478

[B54] Florio M. Huttner W. B. (2014). Neural progenitors, neurogenesis and the evolution of the neocortex. *Development* 141 2182–2194 10.1242/dev.090571141/11/2182 24866113

[B55] Fogarty M. Grist M. Gelman D. Marin O. Pachnis V. Kessaris N. (2007). Spatial genetic patterning of the embryonic neuroepithelium generates GABAergic interneuron diversity in the adult cortex. *J. Neurosci.* 27 10935–10946 10.1523/JNEUROSCI.1629-07.2007 17928435 PMC6672847

[B56] Free S. L. Mitchell T. N. Williamson K. A. Churchill A. J. Shorvon S. D. Moore A. T. (2003). Quantitative MR image analysis in subjects with defects in the PAX6 gene. *Neuroimage* 20 2281–2290 10.1016/j.neuroimage.2003.07.001 14683729

[B57] Fukushima Y. Hoovers J. Mannens M. Wakui K. Ohashi H. Ohno T. (1993). Detection of a cryptic paracentric inversion within band 11p13 in familial aniridia by fluorescence in situ hybridization. *Hum. Genet.* 91 205–209 10.1007/BF00218257 8478003

[B58] Gal J. S. Morozov Y. M. Ayoub A. E. Chatterjee M. Rakic P. Haydar T. F. (2006). Molecular and morphological heterogeneity of neural precursors in the mouse neocortical proliferative zones. *J. Neurosci.* 26 1045–1056 10.1523/JNEUROSCI.4499-05.2006 16421324 PMC3249619

[B59] Gelman D. M. Marin O. (2010). Generation of interneuron diversity in the mouse cerebral cortex. *Eur. J. Neurosci.* 31 2136–2141 10.1111/j.1460-9568.2010.07267.x 20529125

[B60] Gelman D. M. Martini F. J. Nobrega-Pereira S. Pierani A. Kessaris N. Marin O. (2009). The embryonic preoptic area is a novel source of cortical GABAergic interneurons. *J. Neurosci.* 29 9380–9389 10.1523/JNEUROSCI.0604-09.2009 19625528 PMC6665570

[B61] Georgala P. A. Carr C. B. Price D. J. (2011a). The role of Pax6 in forebrain development. *Dev. Neurobiol.* 71 690–709 10.1002/dneu.20895 21538923

[B62] Georgala P. A. Manuel M. Price D. J. (2011b). The generation of superficial cortical layers is regulated by levels of the transcription factor Pax6. *Cereb. Cortex* 21 81–94 10.1093/cercor/bhq061 20413449 PMC3000564

[B63] Gillies K. Price D. J. (1993). The fates of cells in the developing cerebral cortex of normal and methylazoxymethanol acetate-lesioned mice. *Eur. J. Neurosci.* 5 73–84 10.1111/j.1460-9568.1993.tb00207.x 8261092

[B64] Glaser T. Jepeal L. Edwards J. G. Young S. R. Favor J. Maas R. L. (1994). PAX6 gene dosage effect in a family with congenital cataracts, aniridia, anophthalmia and central nervous system defects. *Nat. Genet.* 7 463–471 10.1038/ng0894-463 7951315

[B65] Glaser T. Walton D. S. Maas R. L. (1992). Genomic structure, evolutionary conservation and aniridia mutations in the human PAX6 gene. *Nat. Genet.* 2 232–239 10.1038/ng1192-232 1345175

[B66] Gotz M. Stoykova A. Gruss P. (1998). Pax6 controls radial glia differentiation in the cerebral cortex. *Neuron* 21 1031–1044 10.1016/S0896-6273(00)80621-2 9856459

[B67] Grapp M. Teichler S. Kitz J. Dibaj P. Dickel C. Knepel W. (2009). The homeodomain of PAX6 is essential for PAX6-dependent activation of the rat glucagon gene promoter: evidence for a PH0-like binding that induces an active conformation. *Biochim. Biophys. Acta* 1789 403–412 10.1016/j.bbagrm.2009.02.001 19217949

[B68] Griffin C. Kleinjan D. A. Doe B. Van Heyningen V. (2002). New 3’ elements control Pax6 expression in the developing pretectum, neural retina and olfactory region. *Mech. Dev.* 112 89–100 10.1016/S0925-4773(01)00646-3 11850181

[B69] Gruss P. Walther C. (1992). Pax in development. *Cell* 69 719–722 10.1016/0092-8674(92)90281-G 1591773

[B70] Hack M. A. Sugimori M. Lundberg C. Nakafuku M. Gotz M. (2004). Regionalization and fate specification in neurospheres: the role of Olig2 and Pax6. *Mol. Cell. Neurosci.* 25 664–678 10.1016/j.mcn.2003.12.012S1044743103003956 15080895

[B71] Hansen D. V. Lui J. H. Flandin P. Yoshikawa K. Rubenstein J. L. Alvarez-Buylla A. (2013). Non-epithelial stem cells and cortical interneuron production in the human ganglionic eminences. *Nat. Neurosci.* 16 1576–1587 10.1038/nn.3541 24097039 PMC4191718

[B72] Hansen D. V. Lui J. H. Parker P. R. Kriegstein A. R. (2010). Neurogenic radial glia in the outer subventricular zone of human neocortex. *Nature* 464 554–561 10.1038/nature08845 20154730

[B73] Hatten M. E. (2002). New directions in neuronal migration. *Science* 297 1660–1663 10.1126/science.1074572 12215636

[B74] Haubensak W. Attardo A. Denk W. Huttner W. B. (2004). Neurons arise in the basal neuroepithelium of the early mammalian telencephalon: a major site of neurogenesis. *Proc. Natl. Acad. Sci. U.S.A.* 101 3196–3201 10.1073/pnas.0308600100 14963232 PMC365766

[B75] Haubst N. Berger J. Radjendirane V. Graw J. Favor J. Saunders G. F. (2004). Molecular dissection of Pax6 function: the specific roles of the paired domain and homeodomain in brain development. *Development* 131 6131–6140 10.1242/dev.01524 15548580

[B76] Hauck B. Gehring W. J. Walldorf U. (1999). Functional analysis of an eye specific enhancer of the eyeless gene in *Drosophila*. *Proc. Natl. Acad. Sci. U.S.A.* 96 564–569 10.1073/pnas.96.2.564 9892673 PMC15176

[B77] Haydar T. F. Ang E. Jr. Rakic P. (2003). Mitotic spindle rotation and mode of cell division in the developing telencephalon. *Proc. Natl. Acad. Sci. U.S.A.* 100 2890–2895 10.1073/pnas.04379691000437969100 12589023 PMC151436

[B78] Heine P. Dohle E. Bumsted-O’brien K. Engelkamp D. Schulte D. (2008). Evidence for an evolutionary conserved role of homothorax/Meis1/2 during vertebrate retina development. *Development* 135 805–811 10.1242/dev.012088 18216174

[B79] Heins N. Malatesta P. Cecconi F. Nakafuku M. Tucker K. L. Hack M. A. (2002). Glial cells generate neurons: the role of the transcription factor Pax6. *Nat. Neurosci.* 5 308–315 10.1038/nn828 11896398

[B80] Hendry S. H. Schwark H. D. Jones E. G. Yan J. (1987). Numbers and proportions of GABA-immunoreactive neurons in different areas of monkey cerebral cortex. *J. Neurosci.* 7 1503–1519. 3033170 10.1523/JNEUROSCI.07-05-01503.1987 PMC6568832

[B81] Hevner R. F. (2006). From radial glia to pyramidal-projection neuron: transcription factor cascades in cerebral cortex development. *Mol. Neurobiol.* 33 33–50 10.1385/MN:33:1:033 16388109

[B82] Hingorani M. Williamson K. A. Moore A. T. Van Heyningen V. (2009). Detailed ophthalmologic evaluation of 43 individuals with PAX6 mutations. *Invest. Ophthalmol. Vis. Sci.* 50 2581–2590 10.1167/iovs.08-2827 19218613

[B83] Hisa T. Spence S. E. Rachel R. A. Fujita M. Nakamura T. Ward J. M. (2004). Hematopoietic, angiogenic and eye defects in Meis1 mutant animals. *EMBO J.* 23 450–459 10.1038/sj.emboj.7600038 14713950 PMC1271748

[B84] Ho L. Jothi R. Ronan J. L. Cui K. Zhao K. Crabtree G. R. (2009a). An embryonic stem cell chromatin remodeling complex, esBAF, is an essential component of the core pluripotency transcriptional network. *Proc. Natl. Acad. Sci. U.S.A.* 106 5187–5191 10.1073/pnas.0812888106 19279218 PMC2654397

[B85] Ho L. Ronan J. L. Wu J. Staahl B. T. Chen L. Kuo A. (2009b). An embryonic stem cell chromatin remodeling complex, esBAF, is essential for embryonic stem cell self-renewal and pluripotency. *Proc. Natl. Acad. Sci. U.S.A.* 106 5181–5186 10.1073/pnas.0812889106 19279220 PMC2654396

[B86] Holm P. C. Mader M. T. Haubst N. Wizenmann A. Sigvardsson M. Gotz M. (2007). Loss- and gain-of-function analyses reveal targets of Pax6 in the developing mouse telencephalon. *Mol. Cell Neurosci.* 34 99–119 10.1016/j.mcn.2006.10.008 17158062

[B87] Huttner W. B. Kosodo Y. (2005). Symmetric versus asymmetric cell division during neurogenesis in the developing vertebrate central nervous system. *Curr. Opin. Cell Biol.* 17 648–657 10.1016/j.ceb.2005.10.005 16243506

[B88] Jakovcevski I. Mayer N. Zecevic N. (2011). Multiple origins of human neocortical interneurons are supported by distinct expression of transcription factors. *Cereb. Cortex* 21 1771–1782 10.1093/cercor/bhq245 21139075 PMC3138511

[B89] Jang E. S. Goldman J. E. (2011). Pax6 expression is sufficient to induce a neurogenic fate in glial progenitors of the neonatal subventricular zone. *PLoS ONE* 6:e20894 10.1371/journal.pone.0020894 PMC3117849 21698109

[B90] Jaworski C. Sperbeck S. Graham C. Wistow G. (1997). Alternative splicing of Pax6 in bovine eye and evolutionary conservation of intron sequences. *Biochem. Biophys. Res. Commun.* 240 196–202 10.1006/bbrc.1997.7623 9367909

[B91] Jennings B. H. Ish-Horowicz D. (2008). The Groucho/TLE/Grg family of transcriptional co-repressors. *Genome Biol.* 9 205 10.1186/gb-2008-9-1-205gb PMC2395242 18254933

[B92] Jun S. Desplan C. (1996). Cooperative interactions between paired domain and homeodomain. *Development* 122 2639–2650. 8787739 10.1242/dev.122.9.2639

[B93] Kaldis P. Richardson H. E. (2012). When cell cycle meets development. *Development* 139 225–230 10.1242/dev.073288 22129826

[B94] Kamachi Y. Uchikawa M. Tanouchi A. Sekido R. Kondoh H. (2001). Pax6 and SOX2 form a co-DNA-binding partner complex that regulates initiation of lens development. *Genes Dev.* 15 1272–1286 10.1101/gad.887101 11358870 PMC313803

[B95] Kammandel B. Chowdhury K. Stoykova A. Aparicio S. Brenner S. Gruss P. (1999). Distinct cis-essential modules direct the time-space pattern of the Pax6 gene activity. *Dev. Biol.* 205 79–97 10.1006/dbio.1998.9128 9882499

[B96] Kerwin J. Yang Y. Merchan P. Sarma S. Thompson J. Wang X. (2010). The HUDSEN Atlas: a three-dimensional (3D) spatial framework for studying gene expression in the developing human brain. *J. Anat.* 217 289–299 10.1111/j.1469-7580.2010.01290.x 20979583 PMC2967454

[B97] Kiecker C. Lumsden A. (2005). Compartments and their boundaries in vertebrate brain development. *Nat. Rev. Neurosci.* 6 553–564 10.1038/nrn1702 15959467

[B98] Kim J. Lauderdale J. D. (2006). Analysis of Pax6 expression using a BAC transgene reveals the presence of a paired-less isoform of Pax6 in the eye and olfactory bulb. *Dev. Biol.* 292 486–505 10.1016/j.ydbio.2005.12.041 16464444

[B99] Kiselev Y. Eriksen T. E. Forsdahl S. Nguyen L. H. Mikkola I. (2012). 3T3 cell lines stably expressing Pax6 or Pax6(5a)–a new tool used for identification of common and isoform specific target genes. *PLoS ONE* 7:e31915 10.1371/journal.pone.0031915 PMC3285655 22384097

[B100] Kleinjan D. A. Seawright A. Childs A. J. Van Heyningen V. (2004). Conserved elements in Pax6 intron 7 involved in (auto)regulation and alternative transcription. *Dev. Biol.* 265 462–477 10.1016/j.ydbio.2003.09.011 14732405

[B101] Kleinjan D. A. Seawright A. Elgar G. Van Heyningen V. (2002). Characterization of a novel gene adjacent to PAX6 revealing synteny conservation with functional significance. *Mamm. Genome* 13 102–107 10.1007/s00335-001-3058-y 11889558

[B102] Kleinjan D. A. Seawright A. Mella S. Carr C. B. Tyas D. A. Simpson T. I. (2006). Long-range downstream enhancers are essential for Pax6 expression. *Dev. Biol.* 299 563–581 10.1016/j.ydbio.2006.08.060 17014839 PMC2386664

[B103] Kleinjan D. A. Seawright A. Schedl A. Quinlan R. A. Danes S. Van Heyningen V. (2001). Aniridia-associated translocations, DNase hypersensitivity, sequence comparison and transgenic analysis redefine the functional domain of PAX6. *Hum. Mol. Genet.* 10 2049–2059 10.1093/hmg/10.19.2049 11590122

[B104] Knoepfler P. S. Bergstrom D. A. Uetsuki T. Dac-Korytko I. Sun Y. H. Wright W. E. (1999). A conserved motif N-terminal to the DNA-binding domains of myogenic bHLH transcription factors mediates cooperative DNA binding with pbx-Meis1/Prep1. *Nucleic Acids Res.* 27 3752–3761 10.1093/nar/27.18.3752 10471746 PMC148632

[B105] Kornack D. R. Rakic P. (1998). Changes in cell-cycle kinetics during the development and evolution of primate neocortex. *Proc. Natl. Acad. Sci. U.S.A.* 95 1242–1246 10.1073/pnas.95.3.1242 9448316 PMC18732

[B106] Koroma B. M. Yang J. M. Sundin O. H. (1997). The Pax-6 homeobox gene is expressed throughout the corneal and conjunctival epithelia. *Invest. Ophthalmol. Vis. Sci.* 38 108–120. 9008636

[B107] Kozmik Z. Czerny T. Busslinger M. (1997). Alternatively spliced insertions in the paired domain restrict the DNA sequence specificity of Pax6 and Pax8. *EMBO J.* 16 6793–6803 10.1093/emboj/16.22.6793 9362493 PMC1170283

[B108] Kroll T. T. O’Leary D. D. (2005). Ventralized dorsal telencephalic progenitors in Pax6 mutant mice generate GABA interneurons of a lateral ganglionic eminence fate. *Proc. Natl. Acad. Sci. U.S.A.* 102 7374–7379 10.1073/pnas.0500819102 15878992 PMC1129108

[B109] Krubitzer L. Kaas J. (2005). The evolution of the neocortex in mammals: how is phenotypic diversity generated? *Curr. Opin. Neurobiol.* 15 444–453 10.1016/j.conb.2005.07.003 16026978

[B110] Lauderdale J. D. Wilensky J. S. Oliver E. R. Walton D. S. Glaser T. (2000). 3’ deletions cause aniridia by preventing PAX6 gene expression. *Proc. Natl. Acad. Sci. U.S.A.* 97 13755–13759 10.1073/pnas.240398797 11087823 PMC17648

[B111] Lessard J. Wu J. I. Ranish J. A. Wan M. Winslow M. M. Staahl B. T. (2007). An essential switch in subunit composition of a chromatin remodeling complex during neural development. *Neuron* 55 201–215 10.1016/j.neuron.2007.06.019 17640523 PMC2674110

[B112] Letinic K. Zoncu R. Rakic P. (2002). Origin of GABAergic neurons in the human neocortex. *Nature* 417 645–649 10.1038/nature00779 12050665

[B113] Levers T. E. Edgar J. M. Price D. J. (2001). The fates of cells generated at the end of neurogenesis in developing mouse cortex. *J. Neurobiol.* 48 265–277 10.1002/neu.1056 11500840

[B114] Levitt P. Rakic P. (1980). Immunoperoxidase localization of glial fibrillary acidic protein in radial glial cells and astrocytes of the developing rhesus monkey brain. *J. Comp. Neurol.* 193 815–840 10.1002/cne.901930316 7002963

[B115] Li N. Zhao C. T. Wang Y. Yuan X. B. (2010). The transcription factor Cux1 regulates dendritic morphology of cortical pyramidal neurons. *PLoS ONE* 5:e10596 10.1371/journal.pone.0010596 PMC2868054 20485671

[B116] Long J. E. Cobos I. Potter G. B. Rubenstein J. L. (2009a). Dlx1&2 and Mash1 transcription factors control MGE and CGE patterning and differentiation through parallel and overlapping pathways. *Cereb. Cortex* 19(Suppl. 1) i96–i106 10.1093/cercor/bhp045bhp045 19386638 PMC2693539

[B117] Long J. E. Swan C. Liang W. S. Cobos I. Potter G. B. Rubenstein J. L. (2009b). Dlx1&2 and Mash1 transcription factors control striatal patterning and differentiation through parallel and overlapping pathways. *J. Comp. Neurol.* 512 556–572 10.1002/cne.21854 19030180 PMC2761428

[B118] Lukaszewicz A. Savatier P. Cortay V. Giroud P. Huissoud C. Berland M. (2005). G1 phase regulation, area-specific cell cycle control, and cytoarchitectonics in the primate cortex. *Neuron* 47 353–364 10.1016/j.neuron.2005.06.032 16055060 PMC1890568

[B119] Maekawa M. Iwayama Y. Nakamura K. Sato M. Toyota T. Ohnishi T. (2009). A novel missense mutation (Leu46Val) of PAX6 found in an autistic patient. *Neurosci. Lett.* 462 267–271 10.1016/j.neulet.2009.07.021S0304-3940(09)00939-2 19607881

[B120] Malandrini A. Mari F. Palmeri S. Gambelli S. Berti G. Bruttini M. (2001). PAX6 mutation in a family with aniridia, congenital ptosis, and mental retardation. *Clin. Genet.* 60 151–154 10.1034/j.1399-0004.2001.600210.x 11553050

[B121] Malatesta P. Hack M. A. Hartfuss E. Kettenmann H. Klinkert W. Kirchhoff F. (2003). Neuronal or glial progeny: regional differences in radial glia fate. *Neuron* 37 751–764 10.1016/S0896-6273(03)00116-8 12628166

[B122] Malatesta P. Hartfuss E. Gotz M. (2000). Isolation of radial glial cells by fluorescent-activated cell sorting reveals a neuronal lineage. *Development* 127 5253–5263. 11076748 10.1242/dev.127.24.5253

[B123] Malumbres M. Barbacid M. (2001). To cycle or not to cycle: a critical decision in cancer. *Nat. Rev. Cancer* 1 222–231 10.1038/35106065 11902577

[B124] Malumbres M. Barbacid M. (2005). Mammalian cyclin-dependent kinases. *Trends Biochem. Sci.* 30 630–641 10.1016/j.tibs.2005.09.005 16236519

[B125] Mansouri A. Stoykova A. Gruss P. (1994). Pax genes in development. *J. Cell Sci. Suppl.* 18 35–42 10.1242/jcs.1994.Supplement_18.5 7883790

[B126] Manuel M. Georgala P. A. Carr C. B. Chanas S. Kleinjan D. A. Martynoga B. (2007). Controlled overexpression of Pax6 in vivo negatively autoregulates the Pax6 locus, causing cell-autonomous defects of late cortical progenitor proliferation with little effect on cortical arealization. *Development* 134 545–555 10.1242/dev.02764 17202185 PMC2386558

[B127] Marquardt T. Ashery-Padan R. Andrejewski N. Scardigli R. Guillemot F. Gruss P. (2001). Pax6 is required for the multipotent state of retinal progenitor cells. *Cell* 105 43–55 10.1016/S0092-8674(01)00295-1 11301001

[B128] Marshall C. A. Novitch B. G. Goldman J. E. (2005). Olig2 directs astrocyte and oligodendrocyte formation in postnatal subventricular zone cells. *J. Neurosci.* 25 7289–7298 10.1523/JNEUROSCI.1924-05.2005 16093378 PMC6725308

[B129] Martin R. D. (1990). *Primate Origins and Evolution. A Phylogenetic Reconstruction*. London: Chapman & Hall.

[B130] McBride D. J. Buckle A. Van Heyningen V. Kleinjan D. A. (2011). DNaseI hypersensitivity and ultraconservation reveal novel, interdependent long-range enhancers at the complex Pax6 cis-regulatory region. *PLoS ONE* 6:e28616 10.1371/journal.pone.0028616 PMC3248410 22220192

[B131] McConnell S. K. (1995). Strategies for the generation of neuronal diversity in the developing central nervous system. *J. Neurosci.* 15 6987–6998. 7472455 10.1523/JNEUROSCI.15-11-06987.1995 PMC6578081

[B132] Mercader N. Leonardo E. Azpiazu N. Serrano A. Morata G. Martinez C. (1999). Conserved regulation of proximodistal limb axis development by Meis1/Hth. *Nature* 402 425–429 10.1038/46580 10586884

[B133] Mi D. Carr C. B. Georgala P. A. Huang Y. T. Manuel M. N. Jeanes E. (2013a). Pax6 exerts regional control of cortical progenitor proliferation via direct repression of Cdk6 and hypophosphorylation of pRb. *Neuron* 78 269–284 10.1016/j.neuron.2013.02.012S0896-6273(13)00170-0 23622063 PMC3898967

[B134] Mi D. Huang Y. T. Kleinjan D. A. Mason J. O. Price D. J. (2013b). Identification of genomic regions regulating Pax6 expression in embryonic forebrain using YAC reporter transgenic mouse lines. *PLoS ONE* 8:e80208 10.1371/journal.pone.0080208PONE-D-13-29798 PMC3819282 24223221

[B135] Mikkola I. Bruun J. A. Holm T. Johansen T. (2001). Superactivation of Pax6-mediated transactivation from paired domain-binding sites by dna-independent recruitment of different homeodomain proteins. *J. Biol. Chem.* 276 4109–4118 10.1074/jbc.M008882200 11069920

[B136] Mitchell T. N. Free S. L. Williamson K. A. Stevens J. M. Churchill A. J. Hanson I. M. (2003). Polymicrogyria and absence of pineal gland due to PAX6 mutation. *Ann. Neurol.* 53 658–663 10.1002/ana.10576 12731001

[B137] Miyata T. Kawaguchi A. Okano H. Ogawa M. (2001). Asymmetric inheritance of radial glial fibers by cortical neurons. *Neuron* 31 727–741 10.1016/S0896-6273(01)00420-2 11567613

[B138] Miyata T. Kawaguchi A. Saito K. Kawano M. Muto T. Ogawa M. (2004). Asymmetric production of surface-dividing and non-surface-dividing cortical progenitor cells. *Development* 131 3133–3145 10.1242/dev.01173 15175243

[B139] Miyoshi G. Hjerling-Leﬄer J. Karayannis T. Sousa V. H. Butt S. J. Battiste J. (2010). Genetic fate mapping reveals that the caudal ganglionic eminence produces a large and diverse population of superficial cortical interneurons. *J. Neurosci.* 30 1582–1594 10.1523/JNEUROSCI.4515-09.201030/5/1582 20130169 PMC2826846

[B140] Mo Z. Zecevic N. (2008). Is Pax6 critical for neurogenesis in the human fetal brain? *Cereb. Cortex* 18 1455–1465 10.1093/cercor/bhm181 17947347 PMC2670483

[B141] Moens C. B. Selleri L. (2006). Hox cofactors in vertebrate development. *Dev. Biol.* 291 193–206 10.1016/j.ydbio.2005.10.032 16515781

[B142] Morgan R. (2004). Conservation of sequence and function in the Pax6 regulatory elements. *Trends Genet.* 20 283–287 10.1016/j.tig.2004.04.009S0168952504001076 15219391

[B143] Moskow J. J. Bullrich F. Huebner K. Daar I. O. Buchberg A. M. (1995). Meis1 a PBX1-related homeobox gene involved in myeloid leukemia in BXH-2 mice. *Mol. Cell Biol.* 15 5434–5443. 7565694 10.1128/mcb.15.10.5434 PMC230793

[B144] Muzio L. Dibenedetto B. Stoykova A. Boncinelli E. Gruss P. Mallamaci A. (2002). Emx2 and Pax6 control regionalization of the pre-neuronogenic cortical primordium. *Cereb. Cortex* 12 129–139 10.1093/cercor/12.2.129 11739261

[B145] Muzio L. Mallamaci A. (2003). Emx1 emx2 and pax6 in specification, regionalization and arealization of the cerebral cortex. *Cereb. Cortex* 13 641–647 10.1093/cercor/13.6.641 12764040

[B146] Ninkovic J. Steiner-Mezzadri A. Jawerka M. Akinci U. Masserdotti G. Petricca S. (2013). The BAF complex interacts with Pax6 in adult neural progenitors to establish a neurogenic cross-regulatory transcriptional network. *Cell Stem Cell* 13 403–418 10.1016/j.stem.2013.07.002S1934-5909(13)00310-X 23933087 PMC4098720

[B147] Noctor S. C. Flint A. C. Weissman T. A. Dammerman R. S. Kriegstein A. R. (2001). Neurons derived from radial glial cells establish radial units in neocortex. *Nature* 409 714–720 10.1038/35055553 11217860

[B148] Noctor S. C. Martinez-Cerdeno V. Ivic L. Kriegstein A. R. (2004). Cortical neurons arise in symmetric and asymmetric division zones and migrate through specific phases. *Nat. Neurosci.* 7 136–144 10.1038/nn1172nn1172 14703572

[B149] Oikawa T. Yamada T. (2003). Molecular biology of the Ets family of transcription factors. *Gene* 303 11–34 10.1016/S0378-1119(02)01156-3 12559563

[B150] O’Leary D. D. Chou S. J. Sahara S. (2007). Area patterning of the mammalian cortex. *Neuron* 56 252–269 10.1016/j.neuron.2007.10.010 17964244

[B151] Osumi N. Shinohara H. Numayama-Tsuruta K. Maekawa M. (2008). Concise review: Pax6 transcription factor contributes to both embryonic and adult neurogenesis as a multifunctional regulator. *Stem Cells* 26 1663–1672 10.1634/stemcells.2007-08842007-0884 18467663

[B152] Ouyang J. Shen Y. C. Yeh L. K. Li W. Coyle B. M. Liu C. Y. (2006). Pax6 overexpression suppresses cell proliferation and retards the cell cycle in corneal epithelial cells. *Invest. Ophthalmol. Vis. Sci.* 47 2397–2407 10.1167/iovs.05-1083 16723449

[B153] Paige S. L. Thomas S. Stoick-Cooper C. L. Wang H. Maves L. Sandstrom R. (2012). A temporal chromatin signature in human embryonic stem cells identifies regulators of cardiac development. *Cell* 151 221–232 10.1016/j.cell.2012.08.027S0092-8674(12)01058 22981225 PMC3462257

[B154] Petanjek Z. Dujmovic A. Kostovic I. Esclapez M. (2008). Distinct origin of GABA-ergic neurons in forebrain of man, nonhuman primates and lower mammals. *Coll. Antropol.* 32(Suppl. 1) 9–17. 18405052

[B155] Philips G. T. Stair C. N. Young Lee H. Wroblewski E. Berberoglu M. A. Brown N. L. (2005). Precocious retinal neurons: Pax6 controls timing of differentiation and determination of cell type. *Dev. Biol.* 279 308–321 10.1016/j.ydbio.2004.12.018 15733660 PMC4128400

[B156] Pinson J. Simpson T. I. Mason J. O. Price D. J. (2006). Positive autoregulation of the transcription factor Pax6 in response to increased levels of either of its major isoforms, Pax6 or Pax6(5a), in cultured cells. *BMC Dev. Biol.* 6:25 10.1186/1471-213X-6-25 PMC1489926 16725027

[B157] Plaza S. Aumercier M. Bailly M. Dozier C. Saule S. (1999a). Involvement of poly (ADP-ribose)-polymerase in the Pax-6 gene regulation in neuroretina. *Oncogene* 18 1041–1051 10.1038/sj.onc.1202406 10023680

[B158] Plaza S. Saule S. Dozier C. (1999b). High conservation of cis-regulatory elements between quail and human for the Pax-6 gene. *Dev. Genes Evol.* 209 165–173 10.1007/s004270050240 10079359

[B159] Polyak K. Lee M. H. Erdjument-Bromage H. Koff A. Roberts J. M. Tempst P. (1994). Cloning of p27Kip1 a cyclin-dependent kinase inhibitor and a potential mediator of extracellular antimitogenic signals. *Cell* 78 59–66 10.1016/0092-8674(94)90572-X 8033212

[B160] Price D. J. Aslam S. Tasker L. Gillies K. (1997). Fates of the earliest generated cells in the developing murine neocortex. *J. Comp. Neurol.* 377 414–422 10.1002/(SICI)1096-9861(19970120)377:3<414::AID-CNE8>3.0.CO;2-5 8989655

[B161] Puschel A. W. Gruss P. Westerfield M. (1992). Sequence and expression pattern of pax-6 are highly conserved between zebrafish and mice. *Development* 114 643–651. 1352238 10.1242/dev.114.3.643

[B162] Quinn J. C. Molinek M. Martynoga B. S. Zaki P. A. Faedo A. Bulfone A. (2007). Pax6 controls cerebral cortical cell number by regulating exit from the cell cycle and specifies cortical cell identity by a cell autonomous mechanism. *Dev. Biol.* 302 50–65 10.1016/j.ydbio.2006.08.035 16979618 PMC2384163

[B163] Rakic P. (1974). Neurons in rhesus monkey visual cortex: systematic relation between time of origin and eventual disposition. *Science* 183 425–427 10.1126/science.183.4123.425 4203022

[B164] Rakic P. (1988). Specification of cerebral cortical areas. *Science* 241 170–176 10.1126/science.3291116 3291116

[B165] Richardson J. Cvekl A. Wistow G. (1995). Pax-6 is essential for lens-specific expression of zeta-crystallin. *Proc. Natl. Acad. Sci. U.S.A.* 92 4676–4680 10.1073/pnas.92.10.4676 7753863 PMC42007

[B166] Salomoni P. Calegari F. (2010). Cell cycle control of mammalian neural stem cells: putting a speed limit on G1. *Trends Cell Biol.* 20 233–243 10.1016/j.tcb.2010.01.006 20153966

[B167] Sansom S. N. Griffiths D. S. Faedo A. Kleinjan D. J. Ruan Y. Smith J. (2009). The level of the transcription factor Pax6 is essential for controlling the balance between neural stem cell self-renewal and neurogenesis. *PLoS Genet.* 5:e1000511 10.1371/journal.pgen.1000511 PMC2686252 19521500

[B168] Sansom S. N. Livesey F. J. (2009). Gradients in the brain: the control of the development of form and function in the cerebral cortex. *Cold Spring Harb. Perspect. Biol.* 1 a002519. 10.1101/cshperspect.a002519 PMC2742095 20066088

[B169] Scardigli R. Baumer N. Gruss P. Guillemot F. Le Roux I. (2003). Direct and concentration-dependent regulation of the proneural gene Neurogenin2 by Pax6. *Development* 130 3269–3281 10.1242/dev.00539 12783797

[B170] Schedl A. Ross A. Lee M. Engelkamp D. Rashbass P. Van Heyningen V. (1996). Influence of PAX6 gene dosage on development: overexpression causes severe eye abnormalities. *Cell* 86 71–82 10.1016/S0092-8674(00)80078-1 8689689

[B171] Schmahl W. Knoedlseder M. Favor J. Davidson D. (1993). Defects of neuronal migration and the pathogenesis of cortical malformations are associated with Small eye (Sey) in the mouse, a point mutation at the Pax-6-locus. *Acta Neuropathol. (Berl.)* 86 126–135 10.1007/BF00334879 8213068

[B172] Schmidt-Sidor B. Szymanska K. Williamson K. Van Heyningen V. Roszkowski T. Wierzba-Bobrowicz T. (2009). Malformations of the brain in two fetuses with a compound heterozygosity for two PAX6 mutations. *Folia Neuropathol.* 47 372–382. 20054790

[B173] Schuurmans C. Armant O. Nieto M. Stenman J. M. Britz O. Klenin N. (2004). Sequential phases of cortical specification involve Neurogenin-dependent and -independent pathways. *EMBO J.* 23 2892–2902 10.1038/sj.emboj.7600278 15229646 PMC514942

[B174] Sheng G. Thouvenot E. Schmucker D. Wilson D. S. Desplan C. (1997). Direct regulation of rhodopsin 1 by Pax-6/eyeless in *Drosophila*: evidence for a conserved function in photoreceptors. *Genes Dev.* 11 1122–1131 10.1101/gad.11.9.1122 9159393

[B175] Sherr C. J. Roberts J. M. (1995). Inhibitors of mammalian G1 cyclin-dependent kinases. *Genes Dev.* 9 1149–1163 10.1101/gad.9.10.1149 7758941

[B176] Shimizu N. Watanabe H. Kubota J. Wu J. Saito R. Yokoi T. (2009). Pax6-5a promotes neuronal differentiation of murine embryonic stem cells. *Biol. Pharm. Bull.* 32 999–1003 10.1248/bpb.32.999 19483305

[B177] Shimoda Y. Tajima Y. Osanai T. Katsume A. Kohara M. Kudo T. (2002). Pax6 controls the expression of Lewis x epitope in the embryonic forebrain by regulating alpha 13-fucosyltransferase IX expression. *J. Biol. Chem.* 277 2033–2039 10.1074/jbc.M108495200 11675393

[B178] Singh S. Chao L. Y. Mishra R. Davies J. Saunders G. F. (2001). Missense mutation at the C-terminus of PAX6 negatively modulates homeodomain function. *Hum. Mol. Genet.* 10 911–918 10.1093/hmg/10.9.911 11309364

[B179] Singh S. Stellrecht C. M. Tang H. K. Saunders G. F. (2000). Modulation of PAX6 homeodomain function by the paired domain. *J. Biol. Chem.* 275 17306–17313 10.1074/jbc.M000359200 10747901

[B180] Singh S. Tang H. K. Lee J. Y. Saunders G. F. (1998). Truncation mutations in the transactivation region of PAX6 result in dominant-negative mutants. *J. Biol. Chem.* 273 21531–21541 10.1074/jbc.273.34.21531 9705283

[B181] Singhal N. Graumann J. Wu G. Arauzo-Bravo M. J. Han D. W. Greber B. (2010). Chromatin-remodeling components of the BAF complex facilitate reprogramming. *Cell* 141 943–955 10.1016/j.cell.2010.04.037S0092-8674(10)00491-5 20550931

[B182] Sisodiya S. M. Free S. L. Williamson K. A. Mitchell T. N. Willis C. Stevens J. M. (2001). PAX6 haploinsufficiency causes cerebral malformation and olfactory dysfunction in humans. *Nat. Genet.* 28 214–216 10.1038/90042 11431688

[B183] Skogh C. Eriksson C. Kokaia M. Meijer X. C. Wahlberg L. U. Wictorin K. (2001). Generation of regionally specified neurons in expanded glial cultures derived from the mouse and human lateral ganglionic eminence. *Mol. Cell. Neurosci.* 17 811–820 10.1006/mcne.2001.0973S1044-7431(01)90973-X 11358480

[B184] Smart I. H. Dehay C. Giroud P. Berland M. Kennedy H. (2002). Unique morphological features of the proliferative zones and postmitotic compartments of the neural epithelium giving rise to striate and extrastriate cortex in the monkey. *Cereb. Cortex* 12 37–53 10.1093/cercor/12.1.37 11734531 PMC1931430

[B185] Solomon B. D. Pineda-Alvarez D. E. Balog J. Z. Hadley D. Gropman A. L. Nandagopal R. (2009). Compound heterozygosity for mutations in PAX6 in a patient with complex brain anomaly, neonatal diabetes mellitus, and microophthalmia. *Am. J. Med. Genet. A* 149A, 2543–2546 10.1002/ajmg.a.33081 19876904 PMC2783496

[B186] Stenman J. Yu R. T. Evans R. M. Campbell K. (2003a). Tlx and Pax6 co-operate genetically to establish the pallio-subpallial boundary in the embryonic mouse telencephalon. *Development* 130 1113–1122 10.1242/dev.00328 12571103

[B187] Stenman J. M. Wang B. Campbell K. (2003b). Tlx controls proliferation and patterning of lateral telencephalic progenitor domains. *J. Neurosci.* 23 10568–10576. 14627641 10.1523/JNEUROSCI.23-33-10568.2003 PMC6740920

[B188] Stiles J. Jernigan T. L. (2010). The basics of brain development. *Neuropsychol. Rev.* 20 327–348 10.1007/s11065-010-9148-4 21042938 PMC2989000

[B189] Stoykova A. Gruss P. (1994). Roles of Pax-genes in developing and adult brain as suggested by expression patterns. *J. Neurosci.* 14 1395–1412. 8126546 10.1523/JNEUROSCI.14-03-01395.1994 PMC6577564

[B190] Stoykova A. Treichel D. Hallonet M. Gruss P. (2000). Pax6 modulates the dorsoventral patterning of the mammalian telencephalon. *J. Neurosci.* 20 8042–8050. 11050125 10.1523/JNEUROSCI.20-21-08042.2000 PMC6772738

[B191] Takahashi T. Nowakowski R. S. Caviness V. S. Jr. (1993). Cell cycle parameters and patterns of nuclear movement in the neocortical proliferative zone of the fetal mouse. *J. Neurosci.* 13 820–833. 8426239 10.1523/JNEUROSCI.13-02-00820.1993 PMC6576626

[B192] Takahashi T. Nowakowski R. S. Caviness V. S. Jr. (1995). The cell cycle of the pseudostratified ventricular epithelium of the embryonic murine cerebral wall. *J. Neurosci.* 15 6046–6057. 7666188 10.1523/JNEUROSCI.15-09-06046.1995 PMC6577667

[B193] Tan X. Shi S. H. (2013). Neocortical neurogenesis and neuronal migration. *Wiley Interdiscip. Rev. Dev. Biol.* 2 443–459 10.1002/wdev.88 24014417 PMC3767922

[B194] Tang H. K. Singh S. Saunders G. F. (1998). Dissection of the transactivation function of the transcription factor encoded by the eye developmental gene PAX6. *J. Biol. Chem.* 273 7210–7221 10.1074/jbc.273.13.7210 9516413

[B195] Tarabykin V. Stoykova A. Usman N. Gruss P. (2001). Cortical upper layer neurons derive from the subventricular zone as indicated by Svet1 gene expression. *Development* 128 1983–1993. 11493521 10.1242/dev.128.11.1983

[B196] Tominaga T. Meng W. Togashi K. Urano H. Alberts A. S. Tominaga M. (2002). The Rho GTPase effector protein, mDia, inhibits the DNA binding ability of the transcription factor Pax6 and changes the pattern of neurite extension in cerebellar granule cells through its binding to Pax6. *J. Biol. Chem.* 277 47686–47691 10.1074/jbc.M207539200M207539200 12324464

[B197] Ton C. C. Miwa H. Saunders G. F. (1992). Small eye (Sey): cloning and characterization of the murine homolog of the human aniridia gene. *Genomics* 13 251–256 10.1016/0888-7543(92)90239-O 1612585

[B198] Toresson H. Potter S. S. Campbell K. (2000). Genetic control of dorsal-ventral identity in the telencephalon: opposing roles for Pax6 and Gsh2. *Development* 127 4361–4371. 11003836 10.1242/dev.127.20.4361

[B199] Tuoc T. C. Boretius S. Sansom S. N. Pitulescu M. E. Frahm J. Livesey F. J. (2013a). Chromatin regulation by BAF170 controls cerebral cortical size and thickness. *Dev. Cell* 25 256–269 10.1016/j.devcel.2013.04.005S1534-5807(13)00196-2 23643363

[B200] Tuoc T. C. Narayanan R. Stoykova A. (2013b). BAF chromatin remodeling complex: cortical size regulation and beyond. *Cell Cycle* 12 2953–2959 10.4161/cc.2599925999 23974113 PMC3875669

[B201] Tuoc T. C. Radyushkin K. Tonchev A. B. Pinon M. C. Ashery-Padan R. Molnar Z. (2009). Selective cortical layering abnormalities and behavioral deficits in cortex-specific Pax6 knock-out mice. *J. Neurosci.* 29 8335–8349 10.1523/JNEUROSCI.5669-08.2009 19571125 PMC6665651

[B202] Tuoc T. C. Stoykova A. (2008a). Er81 is a downstream target of Pax6 in cortical progenitors. *BMC Dev. Biol.* 8:23 10.1186/1471-213X-8-231471-213X-8-23 PMC2275226 18307776

[B203] Tuoc T. C. Stoykova A. (2008b). Trim11 modulates the function of neurogenic transcription factor Pax6 through ubiquitin-proteosome system. *Genes Dev.* 22 1972–1986 10.1101/gad.47170822/14/1972 18628401 PMC2492742

[B204] Vidal A. Koff A. (2000). Cell-cycle inhibitors: three families united by a common cause. *Gene* 247 1–15 10.1016/S0378-1119(00)00092-5 10773440

[B205] Visel A. Bristow J. Pennacchio L. A. (2007). Enhancer identification through comparative genomics. *Semin. Cell Dev. Biol.* 18 140–152 10.1016/j.semcdb.2006.12.014 17276707 PMC1855162

[B206] Walcher T. Xie Q. Sun J. Irmler M. Beckers J. Ozturk T. (2013). Functional dissection of the paired domain of Pax6 reveals molecular mechanisms of coordinating neurogenesis and proliferation. *Development* 140 1123–1136 10.1242/dev.082875140/5/1123 23404109 PMC3583046

[B207] Wang B. Long J. E. Flandin P. Pla R. Waclaw R. R. Campbell K. (2013). Loss of Gsx1 and Gsx2 function rescues distinct phenotypes in Dlx1/2 mutants. *J. Comp. Neurol.* 521 1561–1584 10.1002/cne.23242 23042297 PMC3615175

[B208] Warren N. Caric D. Pratt T. Clausen J. A. Asavaritikrai P. Mason J. O. (1999). The transcription factor, Pax6 is required for cell proliferation and differentiation in the developing cerebral cortex. *Cereb. Cortex* 9 627–635 10.1093/cercor/9.6.627 10498281

[B209] Westerman B. A. Murre C. Oudejans C. B. (2003). The cellular Pax-Hox-helix connection. *Biochim. Biophys. Acta* 1629 1–7 10.1016/j.bbaexp.2003.08.002 14522074

[B210] Williams S. C. Altmann C. R. Chow R. L. Hemmati-Brivanlou A. Lang R. A. (1998). A highly conserved lens transcriptional control element from the Pax-6 gene. *Mech. Dev.* 73 225–229 10.1016/S0925-4773(98)00057-4 9622640

[B211] Wilson D. Sheng G. Lecuit T. Dostatni N. Desplan C. (1993). Cooperative dimerization of paired class homeo domains on DNA. *Genes Dev.* 7 2120–2134 10.1101/gad.7.11.2120 7901121

[B212] Wolf L. V. Yang Y. Wang J. Xie Q. Braunger B. Tamm E. R. (2009). Identification of pax6-dependent gene regulatory networks in the mouse lens. *PLoS ONE* 4:e4159 10.1371/journal.pone.0004159 PMC2612750 19132093

[B213] Wonders C. P. Anderson S. A. (2006). The origin and specification of cortical interneurons. *Nat. Rev. Neurosci.* 7 687–696 10.1038/nrn1954 16883309

[B214] Wu J. I. (2012). Diverse functions of ATP-dependent chromatin remodeling complexes in development and cancer. *Acta Biochim. Biophys. Sin. (Shanghai)* 44 54–69 10.1093/abbs/gmr099gmr099 22194014

[B215] Xie Q. Cvekl A. (2011). The orchestration of mammalian tissue morphogenesis through a series of coherent feed-forward loops. *J. Biol. Chem.* 286 43259–43271 10.1074/jbc.M111.264580 21998302 PMC3234836

[B216] Xie Q. Yang Y. Huang J. Ninkovic J. Walcher T. Wolf L. (2013). Pax6 interactions with chromatin and identification of its novel direct target genes in lens and forebrain. *PLoS ONE* 8:e54507 10.1371/journal.pone.0054507 PMC3544819 23342162

[B217] Xu H. E. Rould M. A. Xu W. Epstein J. A. Maas R. L. Pabo C. O. (1999a). Crystal structure of the human Pax6 paired domain-DNA complex reveals specific roles for the linker region and carboxy-terminal subdomain in DNA binding. *Genes Dev.* 13 1263–1275 10.1101/gad.13.10.1263 10346815 PMC316729

[B218] Xu P. X. Zhang X. Heaney S. Yoon A. Michelson A. M. Maas R. L. (1999b). Regulation of Pax6 expression is conserved between mice and flies. *Development* 126 383–395. 9847251 10.1242/dev.126.2.383

[B219] Yan Z. Wang Z. Sharova L. Sharov A. A. Ling C. Piao Y. (2008). BAF250B-associated SWI/SNF chromatin-remodeling complex is required to maintain undifferentiated mouse embryonic stem cells. *Stem Cells* 26 1155–1165 10.1634/stemcells.2007-08462007-0846 18323406 PMC2409195

[B220] Yip D. J. Corcoran C. P. Alvarez-Saavedra M. Demaria A. Rennick S. Mears A. J. (2012). Snf2l regulates Foxg1-dependent progenitor cell expansion in the developing brain. *Dev. Cell* 22 871–878 10.1016/j.devcel.2012.01.020S1534-5807(12)00054-8 22516202 PMC4580287

[B221] Yoneshima H. Yamasaki S. Voelker C. C. Molnar Z. Christophe E. Audinat E. (2006). Er81 is expressed in a subpopulation of layer 5 neurons in rodent and primate neocortices. *Neuroscience* 137 401–412 10.1016/j.neuroscience.2005.08.075 16289830

[B222] Yun K. Potter S. Rubenstein J. L. (2001). Gsh2 and Pax6 play complementary roles in dorsoventral patterning of the mammalian telencephalon. *Development* 128 193–205. 11124115 10.1242/dev.128.2.193

[B223] Zaki P. A. Quinn J. C. Price D. J. (2003). Mouse models of telencephalic development. *Curr. Opin. Genet. Dev.* 13 423–437 10.1016/S0959-437X(03)00084-4 12888017

[B224] Zecevic N. Chen Y. Filipovic R. (2005). Contributions of cortical subventricular zone to the development of the human cerebral cortex. *J. Comp. Neurol.* 491 109–122 10.1002/cne.20714 16127688 PMC2628573

[B225] Zetterberg A. Larsson O. Wiman K. G. (1995). What is the restriction point? *Curr. Opin. Cell Biol.* 7 835–842 10.1016/0955-0674(95)80067-0 8608014

[B226] Zhang W. Cveklovam K. Oppermann B. Kantorow M. Cvekl A. (2001). Quantitation of PAX6 and PAX6(5a) transcript levels in adult human lens, cornea, and monkey retina. *Mol. Vis.* 7 1–5. 11172136 PMC2831401

[B227] Zhou Y. H. Wu X. Tan F. Shi Y. X. Glass T. Liu T. J. (2005). PAX6 suppresses growth of human glioblastoma cells. *J. Neurooncol.* 71 223–229 10.1007/s11060-004-1720-4 15735909

